# Unified total synthesis of the natural products endiandric acid A, kingianic acid E, and kingianins A, D, and F†
†Electronic supplementary information (ESI) available: Details of experimental procedures, spectroscopic data, and copies of ^1^H and ^13^C NMR spectra. See DOI: 10.1039/c5sc00794a



**DOI:** 10.1039/c5sc00794a

**Published:** 2015-05-07

**Authors:** S. L. Drew, A. L. Lawrence, M. S. Sherburn

**Affiliations:** a Research School of Chemistry , Australian National University , Canberra , ACT 2601 , Australia . Email: michael.sherburn@anu.edu.au

## Abstract

A measure of the strength of a synthetic strategy is its versatility: specifically, whether it allows structurally distinct targets to be prepared. Herein we disclose a unified approach for the total synthesis of natural products of three distinct structural types, all of which occur naturally as racemic mixtures. The point of divergence involves the terminal alkylation of a conjugated tetrayne, and culminates in a significantly shortened synthesis of endiandric acid A (8 steps), the first total synthesis of kingianic acid E (8 steps), and a second-generation synthesis of kingianins A, D, and F (11 steps). Evidence for redox catalysis in the biosynthesis of kingianic acid E is presented.

## Abstract 2

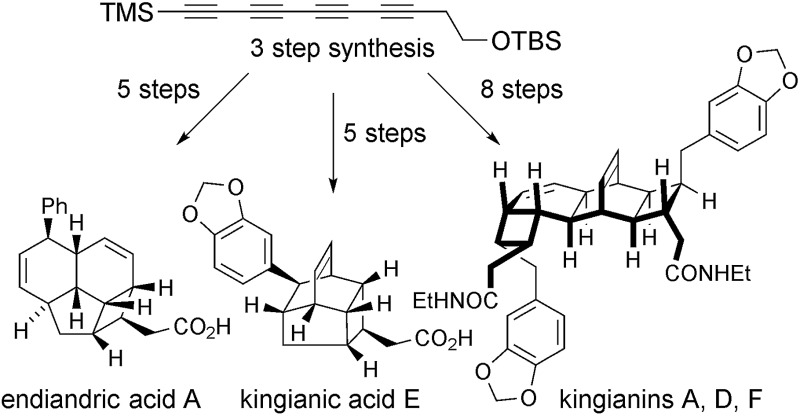
A measure of the strength of a synthetic strategy is its versatility: specifically, whether it allows structurally distinct targets to be prepared. This work describes the total synthesis of natural products of three distinct structural types from a common intermediate.

## Introduction

**Scheme 1:**
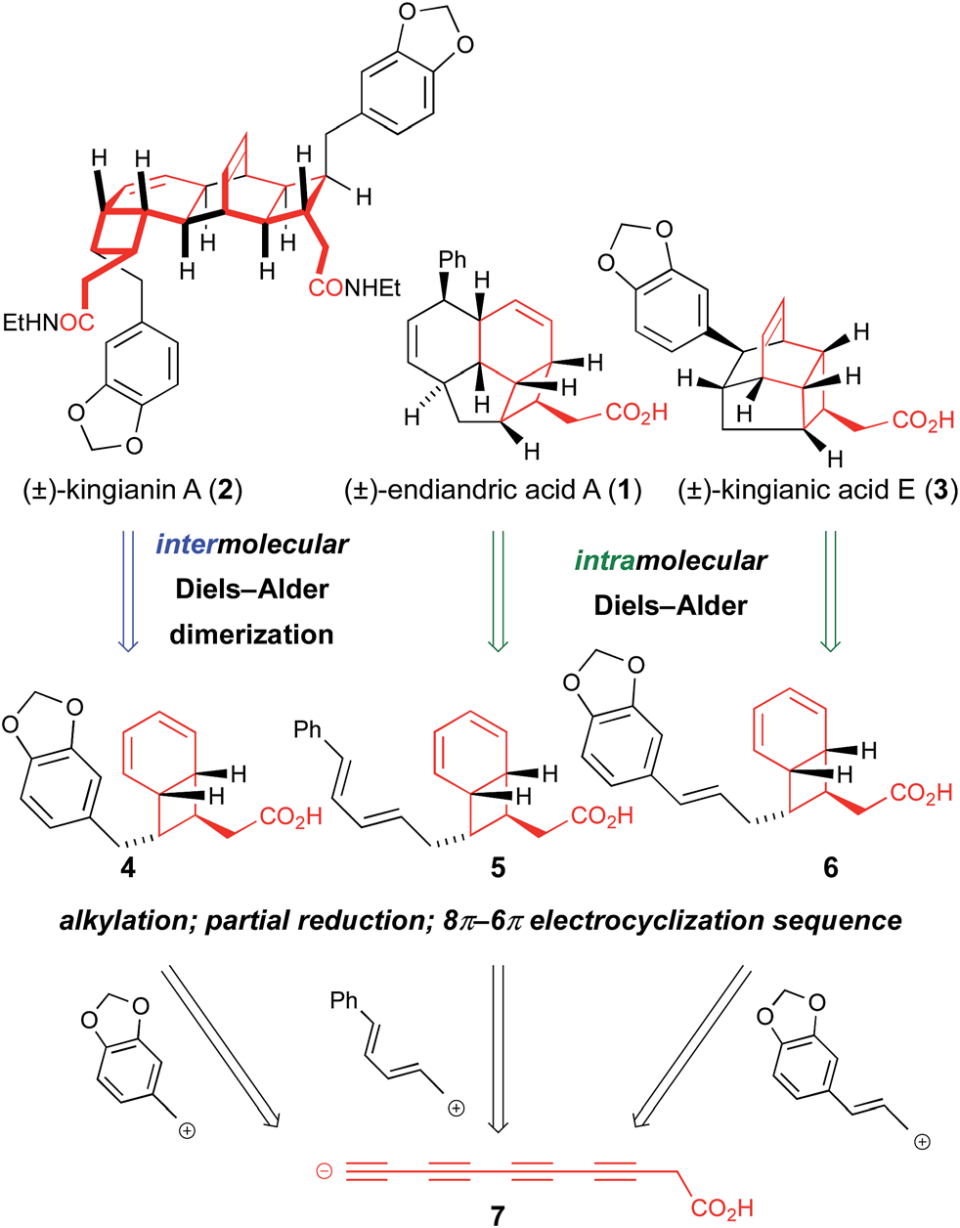
Retrosynthetic analysis of kingianin A, endiandric acid A, and kingianic acid E reveals the potential for a unified total synthesis.

Isolated from the leaves of *Endiandra intorsa*, the endiandric acids^1^ (*e.g.* endiandric acid A, **1**, Scheme 1) possess complex and synthetically challenging tetracyclic frameworks. Black, Banfield and co-workers brilliantly de-convoluted their structural complexity, and proposed a biosynthetic hypothesis involving a domino sequence of pericyclic reactions from linear conjugated (*E*,*Z*,*Z*,*E*)- or (*Z*,*Z*,*Z*,*Z*)-tetraene precursors.^2^ The endiandric acids were isolated as racemic mixtures, hence the proposed thermal 8π-conrotatory/6π-disrotatory electrocyclization-intramolecular Diels–Alder (IMDA) sequence was postulated to occur “readily… in non-enzymic reactions”.^2^ Nicolaou and co-workers' landmark biomimetic synthesis of endiandric acids A–G provided experimental support to the Black/Banfield hypothesis and identified the (*E*,*Z*,*Z*,*E*)-tetraene as a viable biosynthetic precursor.^3^ Our recent total synthesis of the kingianins^4^ (*e.g.* kingianin A, **2**, Scheme 1) – structures that are biosynthetically formulated as products of a thermal 8π–6π electrocyclization then Diels–Alder dimerization – examined the (*Z*,*Z*,*Z*,*Z*)-tetraene as a possible biosynthetic precursor^5^ for the first time.^6^ Thus, a bold approach involving a four-fold *cis*-selective partial reduction of a conjugated tetrayne led to the electrocyclization precursor, albeit from a non-selective crossed Mori–Hiyama coupling. The high temperatures required to achieve (*Z*,*Z*,*Z*,*Z*)-tetraene 8π–6π electrocyclization led us to conclude that (*E*,*Z*,*Z*,*E*)-tetraenes are the more likely biosynthetic precursors to bicyclo[4.2.0]octadiene natural products.^6^ In spite of its unlikely intermediacy in biosynthetic pathways, the deployment of the (*Z*,*Z*,*Z*,*Z*)-tetraene enabled a step-economical^7^ synthesis of kingianins A (**2**), D and F (longest linear sequence 10 steps). Our synthetic studies also demonstrated that the kingianins were likely products of a SET-mediated stepwise intermolecular dimerization process.^6^ Important early studies by Moses described an inability to perform the concerted [4 + 2] cycloaddition.^8*a*^ In studies conducted in parallel with our own, Parker performed an elegant intramolecular SET-mediated dimerization process.^9*a*^ Parker subsequently disclosed a full account of a second-generation approach to kingianin natural products by way of intermolecular formal radical cation Diels–Alder reactions similar to our own.^9*b*^ Most recently, Moses and co-workers successfully prepared kingianin A *via* an electrochemically-mediated formal radical cation Diels–Alder dimerization event.^8*b*^

Recently, Litaudon and co-workers reported the isolation of a second family of natural products from *Endiandra kingiana*, the kingianic acids^10^ (*e.g.* kingianic acid E, **3**, Scheme 1). Biosynthetically, the tetracyclic framework of kingianic acid E (**3**) (Scheme 1) can be viewed as the result of a Black/Banfield-type^2^ domino 8π–6π electrocyclization–IMDA reaction sequence, albeit with diene and dienophile reversed, relative to endiandric acid A. Given the similar biosynthetic origin of kingianin,^4^ endiandric^1^ and kingianic^10^ natural products, we became intrigued by the opportunity for a unified synthetic approach to these polycyclic molecules.

## Results and discussion

At the core of the strategy outlined in Scheme 1 is the identification of a common bicyclo[4.2.0]octadiene–CH_2_CO_2_H motif in precursors **4**, **5**, and **6** to kingianin A (**2**), endiandric acid A (**1**) and kingianic acid E (**3**), respectively. Each precursor would be required to undergo a different mode of (formal) [4 + 2] cycloaddition to furnish the requisite target. Bicyclo[4.2.0]octadienes **4**, **5**, and **6**, differing only in the *endo*-substituent about the cyclobutane ring, could, in principle, be accessed through alkylation of a common tetrayne anion **7** with different electrophiles, followed by four-fold partial reduction and 8π–6π electrocyclization.

**Scheme 2:**
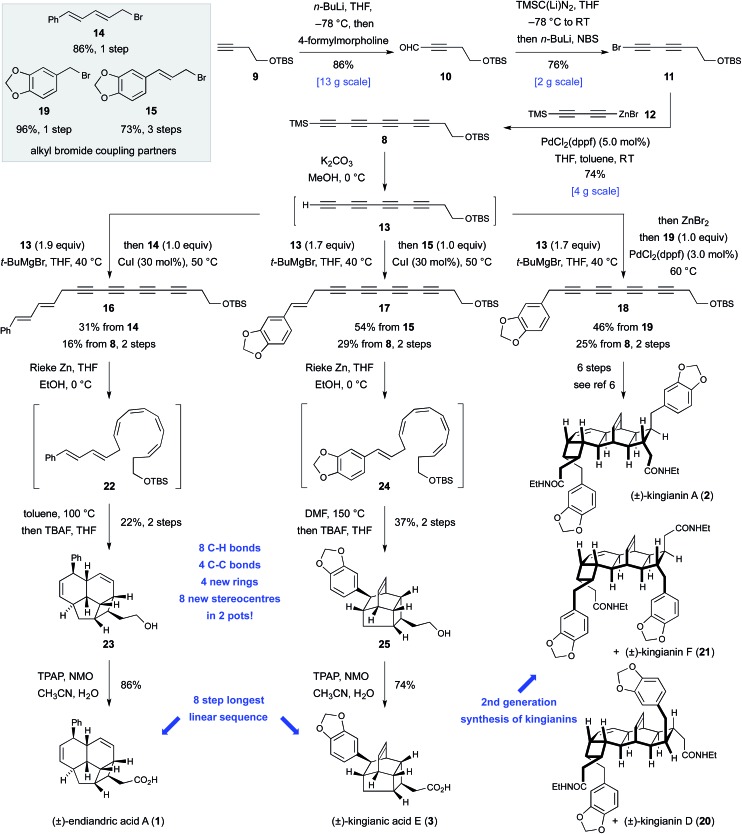
Total synthesis of the racemic natural products endiandric acid A (**1**), kingianic acid E (**3**), and kingianins A (**2**), D (**20**) and F (**21**).

The feasibility of alkylating a synthetic equivalent of tetrayne anion **7** could not be objectively assessed at the outset of this work, since such transformations were without precedent.^11,12^ We viewed this lack of convincing precedent as an opportunity to explore new synthetic space. We elected to target tetrayne **8** (Scheme 2) in anticipation that the TMS- and –CH_2_CH_2_OTBS substituents would bestow a sufficient degree of stabilization to render the compound kinetically stable.^13^ The synthesis of tetrayne **8** was eventually optimized to a three-step sequence from commercially available alkyne **9** (Scheme 2). Thus, deprotonation of terminal alkyne **9** with *n*-butyllithium followed by trapping of the lithium acetylide with 4-formylmorpholine and a phosphate-buffered work-up furnished aldehyde **10** on decagram scale.^14^ Aldehyde **10** was then subjected to a Colvin alkyne homologation,^15^ using lithiated TMS diazomethane, and the resulting terminal diyne was deprotonated and brominated *in situ* to form bromodiyne **11**. This modified homologation protocol, which provides direct access to 1-bromoalkynes from aldehydes, should enjoy wider application.^16^ Negishi cross-coupling^17*a*^ of bromodiyne **11** with organozinc reagent **12** ^17b,c^ delivered tetrayne **8** and set the scene for the point of divergence in the synthesis. This short sequence has been reproduced several times to prepare multi-gram quantities of tetrayne **8**.^18^ All attempts to directly generate an anionic-tetrayne species from TMS tetrayne **8** using TBAF^19^ or MeLi^17*b*^ led to decomposition. This problem was solved by firstly generating the terminal tetrayne **13** ^20^ by selective desilylation of **8** with potassium carbonate in methanol, and subsequent metalation with *tert*-butylmagnesium bromide. The resulting tetrayne Grignard reagent was then alkylated with allylic bromides **14** ^21^ and **15** under Cu(i) catalysis.^22^ Tetraynes **16** and **17** were isolated in 16% and 29% yield, respectively, over two steps from TMS tetrayne **8**. In the case of tetrayne **18**, a Negishi cross coupling protocol, with benzyl bromide **19**, proved superior.^17*a*^ Thus, all of the carbon atoms required for endiandric acid A (**1**), kingianic acid E (**3**), and kingianins A (**2**), D (**20**), and F (**21**) were installed in a longest linear sequence of five steps from commercially available precursors. The somewhat modest yields obtained for the metallotetrayne alkylation betray a seminal transformation that requires further optimization. Nonetheless, the generality of this transformation, and the step-economy^7^ that it conveys upon this synthetic approach, are undeniable. This second-generation synthesis of unsymmetrical tetrayne **18** constitutes a formal synthesis of kingianins A, D, and F, and avoids the use of a non-selective Mori–Hiyama C(sp)–C(sp) cross-coupling, which was a weakness in our previous approach.^6^

Conversion of tetraynes **16** and **17** into endiandric acid A (**1**) and kingianic acid E (**3**) first required transformation of the conjugated tetrayne units into the corresponding (*Z*,*Z*,*Z*,*Z*)-tetraenes. Subjection of endiandric tetrayne **16** to our previously optimized *cis*-selective partial reduction conditions^6^ successfully generated (*Z*,*Z*,*Z*,*Z*)-tetraene **22**. This material was then immediately heated to 100 °C in toluene to bring about the requisite domino 8π–6π electrocyclization–IMDA sequence. Subsequent TBS deprotection, conducted in the same flask, led to isolation of alcohol **23** in 22% yield from tetrayne **16**. Finally, oxidation with TPAP/NMO^23^ furnished a synthetic sample of endiandric acid A (**1**).^24^ Thus, some 30 years after Black and Banfield's biosynthetic hypothesis and Nicolaou's ground-breaking biomimetic synthesis *via* the (*E*,*Z*,*Z*,*E*)-tetraene, the (*Z*,*Z*,*Z*,*Z*)-tetraene route to endiandric acid A has finally been realized in the laboratory.

The first total synthesis of kingianic acid E (**3**) was achieved in a similar fashion. Partial reduction of kingianic tetrayne **17**, followed by heating of (*Z*,*Z*,*Z*,*Z*)-tetraene **24** to 150 °C in DMF and *in situ* TBAF deprotection, led to the isolation of tetracyclic alcohol **25** in 37% yield. Oxidation gave kingianic acid E (**3**), the analytical data of which matched that reported by Litaudon and co-workers in all respects.^10^

**Scheme 3:**
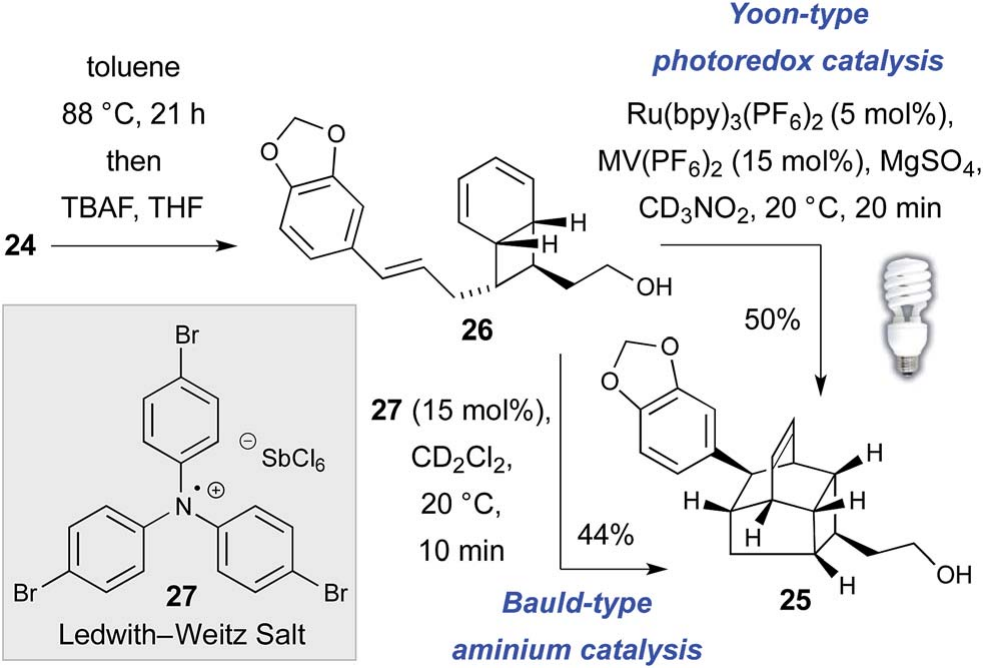
Radical cation formal IMDA reaction of bicyclo[4.2.0]octadiene **26**.

The thermal, concerted IMDA reaction en route to kingianic acid E (**3**) was sufficiently sluggish at 88 °C to allow isolation of bicyclo[4.2.0]octadiene **26** (Scheme 3). The lethargic behavior of this concerted cycloaddition is hardly surprising given the electron rich nature of the dienophile of **26**. In light of this observation, we ventured that the biosynthesis of kingianic acid E (**3**) might involve a redox-catalyzed process.

Support for this proposal was obtained in the form of a rapid formal Diels–Alder reaction of bicyclo[4.2.0]octadiene **26** to alcohol **25** at ambient temperature using either visible light photoredox catalysis^25^ or *via* treatment with the Ledwith–Weitz radical cation salt **27**.^26^ Given that, under redox catalysis, the formal IMDA reaction of bicyclo[4.2.0]octadiene **26** is significantly faster than the concerted, thermal cycloaddition, we became curious as to whether the entire 8π–6π double electrocyclization–IMDA sequence might be a redox-catalyzed process in Nature. While there is no compelling mechanistic case for redox catalysis of the electrocyclization sequence, in principle the conversion of tetraene **24** to bicycle **26** could also occur by way of a redox-catalyzed [2 + 2]-cycloaddition mechanism, for which there is precedent.^27^ Disappointingly, when (*Z*,*Z*,*Z*,*Z*)-tetraene **24** was subjected to both Bauld-type aminium catalysis and Yoon-type photoredox catalysis, only complex mixtures resulted, with no sign of the target products. These results do not disprove redox catalysis of the complete process (*i.e.* from tetraene **24** to caged product **25**) in nature but they do appear to indicate that the formal Diels–Alder process is especially predisposed to it.

## Conclusions

In summary, a unified synthesis of three major sub-families of polycyclic natural products from the *Endiandra* genus of plants has been accomplished. Our recently-developed four-fold *cis*-selective partial reduction of conjugated tetraynes to (*Z*,*Z*,*Z*,*Z*)-tetraenes has inspired a significantly shortened synthesis of endiandric acid A (longest linear sequences: this work, 8 steps; previous syntheses 14 steps [methyl ester]^3^ and 23 steps^28^), the first total synthesis of kingianic acid E and an improved synthesis of three kingianin natural products. We anticipate that this approach will prove applicable to step-economical syntheses of related natural products.
