# Supplementary material for: Unified total synthesis of the natural products endiandric acid A, kingianic acid E, and kingianins A, D, and F
Source: Chem Sci. 2015 May 7;6(7):3886–90. doi: 10.1039/c5sc00794a (PMC5707472; doi:10.1039/c5sc00794a)
Supplement: Supplementary file 1 [file SC-006-C5SC00794A-s001.pdf]

Electronic Supplementary Material (ESI) for Chemical Science.
This journal is © The Royal Society of Chemistry 2015

SUPPORTING INFORMATION

Unified Total Synthesis of the Natural Products Endiandric Acid A, Kingianic Acid E, and Kingianins A, D, and F

Samuel L. Drew, Andrew L. Lawrence,*[a] and Michael S. Sherburn*

Research School of Chemistry, Australian National University, Canberra ACT 2601, Australia

*E-mail: michael.sherburn@anu.edu.au, a.lawrence@ed.ac.uk

[a]Present address: School of Chemistry, University of Edinburgh, Joseph Black Building, West Mains Road, Edinburgh, EH9 3JJ, United Kingdom.

Contents of Supporting Information

General Experimental Methods S3

Experimental Procedures and Characterization Data

Aldehyde 10 S5

Bromodiyne 11 S6

TMS tetrayne 8 S7

Alkyl bromide 14 S8

Alkyl bromide 15 S10

Benzyl bromide 19 S11

Endiandric tetrayne 16 S12

(±)-Alcohol 23 S14

(±)-Endiandric acid A (1) S16

Kingianic tetrayne 17 S17

(±)-Alcohol 25 S19

(±)-Kingianic acid E (3) S21

Kingianin tetrayne 18 S24

(±)-Bicyclo[4.2.0]octadiene 26 S26

Radical cation formal IMDA reaction of (±)-bicyclo[4.2.0]octadiene 26 S28

References S29

^{1}H and ^{13}C NMR Spectra S30

General Experimental Methods

NMR spectra

^{1}H NMR spectra were recorded at 298K using a Bruker AVANCE 800, Bruker AVANCE 600, Bruker AVANCE 400, or Varian 400-MR spectrometer as indicated. Residual monoprotic solvent peaks were used as an internal reference for ^{1}H NMR spectra ($CDCl_3$ δ 7.26, CD_2Cl_2 δ 5.32 ppm). Coupling constants (J) are quoted to the nearest 0.1 Hz. The assignment of proton signals was assisted by COSY, HSQC and HMBC experiments where necessary. ^{13}C NMR spectra were recorded at 298 K using a Bruker AVANCE 800, Bruker AVANCE 600, Bruker AVANCE 400, or Varian 400-MR spectrometer, as indicated. Residual monoprotic solvent peaks were used as an internal reference for ^{13}C NMR spectra ($CDCl_3$ δ 77.16 ppm, CD_2Cl_2 δ 53.84 ppm). Assignment of carbon signals was assisted by COSY, HSQC and HMBC experiments where necessary. The following abbreviations (or combinations thereof) were used to describe ^{1}H NMR multiplicities: s = singlet, d = doublet, t = triplet, q = quartet, m = multiplet, br. = broad.

IR spectra

IR spectra were recorded on a Perkin–Elmer 1600 FTIR spectrometer as potassium bromide discs or as thin films on NaCl plates.

Mass spectrometry

Low-resolution EI mass spectra were recorded on an Agilent HP 6890 series gas GC/MS with a 7683 series injector. High-resolution EI mass spectra were recorded on a Waters AutoSpec Premier spectrometer magnetic sector instrument, operating at 70 eV. Low-resolution ESI mass spectra were recorded on a ZMD Micromass spectrometer with Waters Alliance 2690 HPLC. High-resolution ESI mass spectra were recorded on a Waters LCT Premier time-of-flight (TOF) mass spectrometer.

HPLC

Analytical HPLC was performed using an Agilent 1100 quaternary pump, automatic liquid sampler, column compartment and diode array detector. Preparative HPLC was conducted using an Agilent 1100 preparative binary pump system, preparative automatic liquid sampler, and diode array detector with a preparative flow cell.

Melting points

Melting points were measured on a Stanford Research Systems OptiMelt MPA100.

Analytical TLC

Analytical TLC was performed with Merck silica gel plates, pre-coated with silica gel 60 F254 (0.2 mm). Visualisation was effected by quenching of UV fluorescence (λ_{max}= 254 nm) and by staining with *p*-anisaldehyde or 2,4-dinitrophenylhydrazine TLC stain solutions, followed by heating.

Flash chromatography

Flash chromatography employed Merck Kiesegel 60 (230–400 mesh) silica gel.

Handling of tetraynes and tetraenes

Small quantities of BHT were added during aqueous work ups (typically 1–2 mL of BHT solution containing 1 crystal of BHT in 100 mL extraction solvent) and to solvents during column chromatography (1 crystal per 250–300 mL mobile phase). Light exposure was minimised when handling tetraenes. The tetraynes were found to be acid sensitive and were purified using phosphate buffered SiO_2 gel (pH 7). Reactions involving the preparation or reaction of tetraynes were conducted behind blast shields as a safety precaution.

Experimental procedures, reagents and glassware

Reactions were conducted under a positive pressure of dry nitrogen in oven-dried glassware, and at ambient room temperature, unless specified otherwise. Anhydrous solvents were either obtained from commercial sources or dried according to the procedure outlined by Grubbs and co-workers.[1] Commercially available chemicals were used as purchased, or where specified, purified by standard techniques.[2]

Unified Total Synthesis of the Natural Products Endiandric Acid A, Kingianic Acid E, and Kingianins A, D, and F

Experimental Procedures and Characterization Data

*Three step sequence to TMS tetrayne **8***

Aldehyde 10

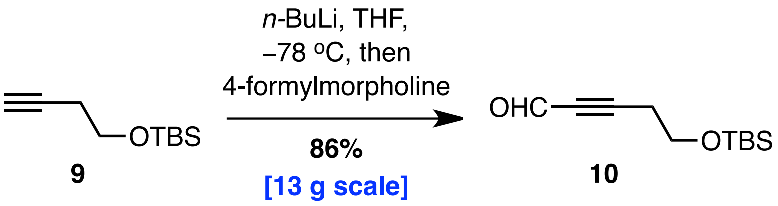

This compound was prepared according to a modified literature procedure.[3] A 3-neck 1 L round-bottomed flask equipped with an equalizing addition funnel was charged with (but-3-yn-1-yloxy)(*tert*-butyl)dimethylsilane **9** (13.1 g, 71.1 mmol, 1.0 mol. equiv.) and dry THF (500 mL). The resulting solution was cooled to −78 °C and *n*-BuLi (1.56 M in hexanes, 54.7 mL, 85.3 mmol, 1.2 mol. equiv.) was transferred by cannula to the addition funnel and added slowly over 5 min. The resulting intense purple solution was stirred for an additional 30 min after which time dry 4-formylmorpholine (10.0 mL, 99.5 mmol, 1.4 mol. equiv.) was added dropwise. The resulting mixture was warmed to ambient room temperature, stirred for 2 h, then poured onto ice-cooled 10% *aq.* KH_2PO_4 solution (200 mL) and diluted with Et_2O (200 mL). The aqueous layer was separated and back extracted with additional Et_2O (200 mL). The organic layers were combined, washed with brine (200 mL), and dried over $MgSO_4$. Concentration under reduced pressure (0 °C, *compound is volatile*) and filtration through a short plug of SiO_2 (CH_2Cl_2) furnished aldehyde **10** as a dark orange oil (13.0 g, 61.3 mmol, 86%). ^1H and ^{13}C NMR spectra matched that previously reported.[3]

Bromodiyne 11

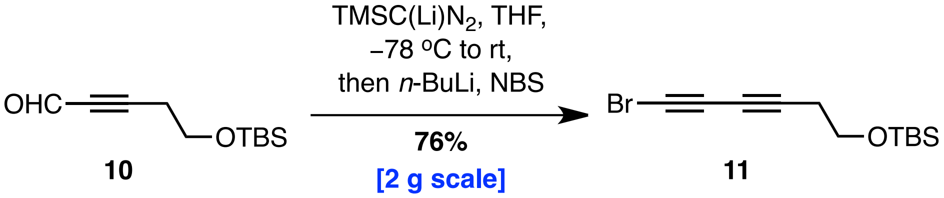

Warning: Appropriate precautions need to be made when handling trimethylsilyldiazomethane. Vapor inhalation has resulted in the death of two researchers in the last decade.

A 3-neck 250 mL round-bottomed flask was charged with TMS diazomethane solution (2.0 M in hexanes, 5.44 mL, 10.9 mmol, 1.1 mol. equiv.) and dry THF (70 mL). The yellow solution was cooled to −78 °C and *n*-BuLi (1.56 M in hexanes, 7.00 mL, 10.9 mmol, 1.1 mol. equiv.) was added. The resulting orange solution was stirred for a further 15 min and then aldehyde **10** (2.10 g, 9.89 mmol, 1.0 mol. equiv.) dissolved in dry THF (10 mL) was added slowly over 5 min. The reaction mixture was then warmed to ambient room temperature and stirred until effervescence had ceased (*evolution of* N_2) and TLC analysis indicated the complete consumption of starting material (*typically 1 h*). The resulting solution, which contained the intermediate terminal diyne, was then re-cooled to −78 °C and a second portion of *n*-BuLi (1.56 M in hexanes, 8.23 mL, 12.8 mmol, 1.3 mol. equiv.) was added, and the mixture stirred for 15 min. *N*-Bromosuccinimde (2.64 g, 14.8 mmol, 1.5 mol. equiv.) was then added and the dark brown reaction mixture was warmed to ambient room temperature and stirred in the dark for 1 h. The reaction was quenched with *sat. aq.* NH_4Cl solution (100 mL) and diluted with Et_2O (100 mL). The aqueous phase was separated and back extracted with Et_2O (100 mL). The organic layers were combined, washed with brine (100 mL), and dried over $MgSO_4$. Concentration under reduced pressure and purification by flash column chromatography (SiO_2, 9:1 petroleum ether 40–60/CH_2Cl_2) afforded the title compound **11** as a pale yellow oil (2.17 g, 7.55 mmol, 76%).

R*f* = 0.65 (2:1 petroleum ether 40–60/CH_2Cl_2);

^{1}H NMR (400 MHz, $CDCl_3$) δ 3.73 (t, *J* = 6.9 Hz, 2H), 2.46 (t, *J* = 6.9 Hz, 2H), 0.89 (s, 9H), 0.07 (s, 6H) ppm;

^{13}C NMR (100 MHz, $CDCl_3$) δ 74.5 (C_q), 66.6 (C_q), 65.8 (C_q), 61.4 (CH_2), 37.5 (C_q), 26.0 (CH_3), 23.4 (CH_2), 18.5 (C_q), –5.2 (CH_3) ppm;

IR (thin film): ν_{max} = 2954, 2929, 2857, 2143 cm^{-1};

MS (EI): *m/z* (%): 286 ($M^{+\bullet}$(^{79}Br), 0 %), 271 ($M^{+\bullet}$(^{79}Br) – CH_3, 5 %), 229 ($M^{+\bullet}$(^{79}Br) – ^{t}Bu, 100 %);

HRMS (EI): calculated for fragment $C_{11}H_{16}OSi^{79}Br$ [$M^{+\bullet}$(^{79}Br) – CH_3]: 271.0154; found 271.0161.

TMS Tetrayne 8

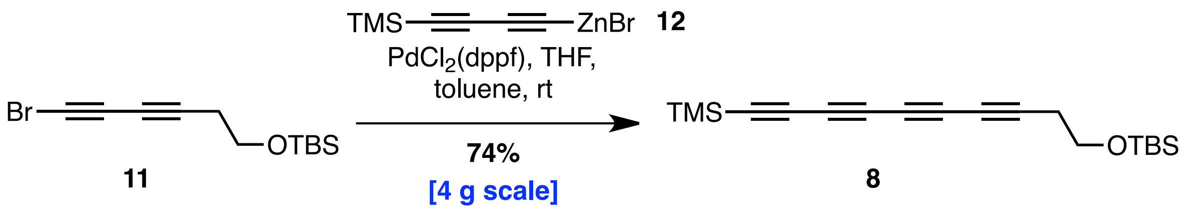

A 3-neck 250 mL round-bottomed flask was charged with 1,4-bis(trimethylsilyl)butadiyne (4.40 g, 22.6 mmol, 1.3 mol. equiv.) and dry THF (25 mL). MeLi.LiBr complex solution (1.5 M in Et_2O, 15.1 mL, 22.6 mmol, 1.3 mol. equiv.) was then added and the resulting purple solution stirred for 30 min at ambient room temperature. After this time the reaction mixture was cooled to 0 °C and dry $ZnBr_2$ solution (1.50 M in THF, 15.1 mL, 22.6 mmol, 1.3 mol. equiv.) was added, and the resulting organozinc reagent **12** stirred at ambient room temperature for an additional 30 min. Bromodiyne **11** (5.00 g, 17.4 mmol, 1.0 mol. equiv.), dry toluene (105 mL), and $PdCl_2$(dppf) (0.637 g, 0.871 mmol, 5.0 mol%) were then added sequentially and the reaction stirred in the dark for a further 18 h. The reaction was quenched with *sat. aq.* NH_4Cl solution (200 mL) and diluted with Et_2O (200 mL). The aqueous phase was separated and back extracted with Et_2O (200 mL). The organic layers were combined, washed with brine (200 mL), and dried over $MgSO_4$. Concentration under reduced pressure and purification by flash column chromatography (SiO_2, 1:0 to 6:1 petroleum ether 40–60/CH_2Cl_2) afforded TMS tetrayne **8** as a dark brown oil (4.23 g, 12.9 mmol, 74%). TMS tetrayne **8** was stored neat in the freezer over a period of months with no apparent decomposition.

R_f = 0.45 (6:1 petroleum ether 40–60/CH_2Cl_2);
^{1}H NMR (400 MHz, $CDCl_3$) δ 3.75 (t, J = 6.8 Hz, 2H), 2.52 (t, J = 6.8 Hz, 2H), 0.89 (s, 9H), 0.21 (s, 9H), 0.072 (s, 6H) ppm;
^{13}C NMR (100 MHz, $CDCl_3$) δ 88.2 (C_q), 87.3 (C_q), 78.6 (C_q), 66.7 (C_q), 62.9 (C_q), 62.7 (C_q), 61.2 (CH_2), 61.1 (C_q), 60.6 (C_q), 26.0 (CH_3), 24.1 (CH_2), 18.4 (C_q), –0.4 (CH_3), –5.2 (CH_3) ppm;
IR (thin film): ν_{max} = 2956, 2929, 2857, 2220, 2144, 2062, 1252, 1113 cm^{-1};
MS (EI): *m/z* (%): 328 ($M^{+\bullet}$, 8 %), 271 ($M^{+\bullet}$– ^{t}Bu, 100 %);
HRMS (EI): calculated for $C_{19}H_{28}OSi_2$ $[M]^{+\bullet}$: 328.1679; found 328.1681.

Preparation of alkyl bromide coupling partners

Alkyl bromide 14

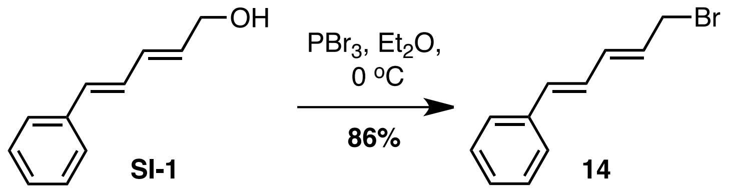

This compound was prepared according to a modified literature procedure.[4] To a 2-neck 50 mL round-bottomed flask was added commercially available alcohol **SI-1** (0.200 g, 1.25 mmol, 1.0 mol. equiv.) and dry Et_2O (5.0 mL). The resulting solution was cooled to 0 °C and PBr_3 (0.13 mL, 1.31 mmol, 1.05 mol. equiv.) was added dropwise. After stirring at 0 °C for 30 min the reaction was quenched with ice-water and diluted with Et_2O (10 mL). The aqueous phase was separated and back extracted with Et_2O (20 mL). The organic layers were combined, washed with *sat. aq.* $NaHCO_3$ solution (10 mL), brine (10 mL), and dried over $MgSO_4$. Concentration under reduced pressure furnished alkyl bromide **14** as a white solid (1.07 mmol by ^1H NMR using durene as an internal standard, 86%). The title compound was found to be unstable on SiO_2 gel and crude material was used in subsequent reactions. ^1H and ^{13}C NMR spectra of **14** matched that previously reported.[4]

Alcohol SI-3

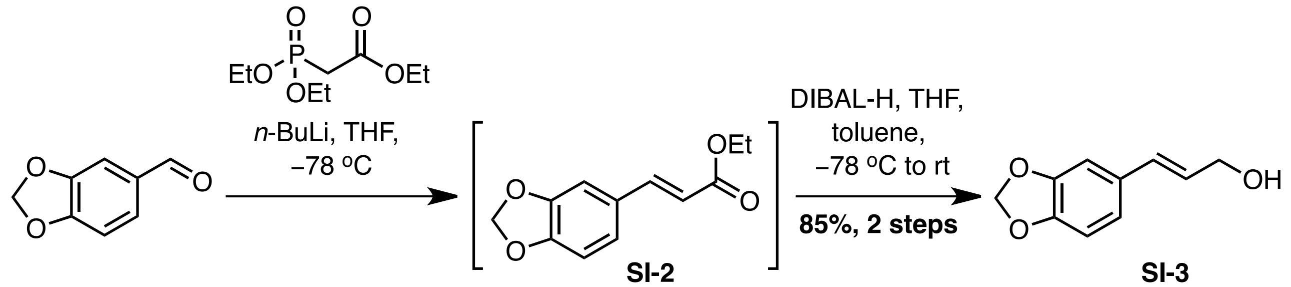

To a 3-neck 500 mL round-bottomed flask was added triethyl phosphonoacetate (8.21 g, 36.6 mmol, 1.1 mol. equiv.) and dry THF (180 mL). The resulting solution was cooled to −78 °C and *n*-BuLi (1.44 M in hexanes, 36.6 mmol, 1.1 mol. equiv.) was added and the reaction stirred for 15 min at −78 °C during which time an orange colour developed. Piperonal (5.00 g, 33.3 mmol, 1.0 mol. equiv.) was then added and the reaction mixture was warmed to ambient room temperature and stirred for 2 h. The reaction was quenched with *sat. aq.* NH_4Cl solution (100 mL) and diluted with Et_2O (100 mL). The aqueous phase was separated and back extracted with Et_2O (100 mL). The organic layers were combined, washed with brine (100 mL), and dried over $MgSO_4$. Concentration under reduced pressure furnished ester **SI-2** (10.0 g crude mass), which was then subjected directly to the next step without further purification.

To a 3-neck 500 mL round-bottomed flask containing ester **SI-2** (10.0 g crude mass) in dry THF (120 mL) was added DIBAL-H (1.0 M in toluene, 83.3 mL, 83.3 mmol, ~2.5 mol. equiv.) slowly over 20 min at −78 °C. Once the addition was complete the reaction mixture was warmed to ambient room temperature and stirred for 4 h. After this time residual DIBAL-H was cautiously quenched with *i*-PrOH → EtOH → MeOH at 0 °C under N_2. The reaction mixture was diluted with Et_2O (50 mL), *sat. aq.* $NaHCO_3$ solution (75 mL), and *sat. aq.* Rochelle's salt solution (75 mL), and stirred for 1 h until two distinct layers had formed. The aqueous phase was separated and back extracted with Et_2O (150 mL). The organic layers were combined, washed with brine (100 mL), and dried over $MgSO_4$. Concentration under reduced pressure and purification by flash column chromatography (SiO_2, 1:1 petroleum ether 40–60/Et_2O) afforded alcohol **SI-3** as a white solid (5.07 g, 28.4 mmol, 85%). ^{1}H and ^{13}C NMR spectra of **SI-3** matched that previously reported.[5]

Alkyl bromide 15

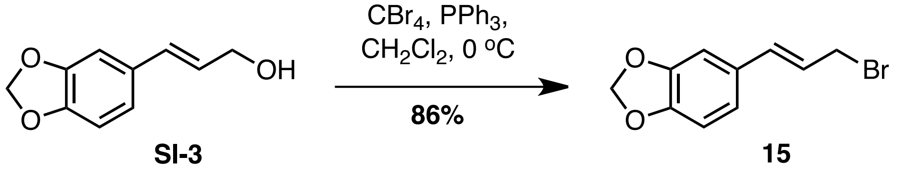

A 3-neck 100 ml round-bottomed flask was charged with alcohol **SI-3** (0.800 g, 4.49 mmol, 1.0 mol. equiv.) and dry CH_2Cl_2 (18.0 mL). The resulting solution was cooled to 0 °C and PPh_3 (1.30 g, 4.96 mmol, 1.10 mol. equiv.) and CBr_4 (1.64 g, 4.96 mmol, 1.10 mol. equiv.) were added and the reaction mixture was stirred for 30 min at 0 °C and then 30 min at ambient room temperature. After this time, ^{1}H NMR analysis of a small aliquot from the reaction mixture indicated that the reaction had reached completion. The reaction mixture was diluted with *n*-pentane (10 mL) and Et_2O (10 mL) and filtered through Celite, rinsing thoroughly with Et_2O. Concentration under reduced pressure furnished alkyl bromide **15** (3.88 mmol by ^{1}H NMR analysis using durene as an internal standard, 86%), which was contaminated with a residual amount of triphenylphosphine oxide. Alkyl bromide **15** was found to be moisture sensitive and unstable, and as such was used without further purification. Attempts to prepare alkyl bromide **15** *via* the PBr_3-mediated protocol used to synthesize alkyl bromide **14** resulted in complex mixtures.

R*f* (unstable on SiO_2 gel);
^{1}H NMR (400 MHz, $CDCl_3$) δ 6.90 (br s, 1H), 6.82 – 6.78 (m, 1H), 6.73 (d, J = 8.0 Hz, 1H), 6.53 (d, J = 15.5 Hz, 1H), 6.20 (dt, J = 15.5, 7.9 Hz, 1H), 5.93 (s, 2H), 4.12 (d, J = 7.8 Hz, 2H) ppm;
^{13}C NMR (100 MHz, $CDCl_3$) δ 148.2 (C_q), 147.9 (C_q), 134.3 (CH), 130.3 (C_q), 123.4 (CH), 121.9 (CH), 108.4 (CH), 105.9 (CH), 101.3 (CH_2), 33.9 (CH_2) ppm;
IR (thin film): ν_{max} = 2895, 1503, 1489, 1445, 1250, 1040 cm^{-1};
MS (EI): *m/z* (%): 239 ($M^{+\bullet}$(^{79}Br), 15 %), 161 ($M^{+\bullet}$(^{79}Br) – (^{79}Br), 100 %);
HRMS (EI): calculated for $C_{10}H_9O_2{}^{79}Br$ $[M]^{+\bullet}$: 239.9786; found 239.9787.

Benzyl bromide 19

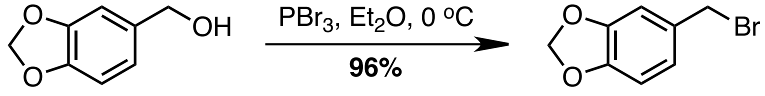

19

This compound, although commercially available, was prepared according to a modified literature procedure.[6] To a 1 L 3-neck round-bottomed flask, equipped with an addition funnel, was added piperonyl alcohol (17.0 g, 112 mmol, 1.0 mol. equiv.) and dry Et_2O (170 mL). The resulting mixture was cooled to 0 °C and the addition funnel charged with PBr_3 (11.7 mL, 123 mmol, 1.1 mol. equiv.) and dry Et_2O (140 mL). The subsequent ethereal-PBr_3 mixture was added slowly to the piperonyl alcohol solution over 15 min. After an additional 30 min, the reaction mixture was quenched with water (300 mL) at 0 °C under N_2. The aqueous layer was separated and back extracted with additional Et_2O (3 × 150 mL). The organic layers were combined, washed with brine (300 mL), and dried over $MgSO_4$. Concentration under reduced pressure yielded the title compound **19** as a colourless oil that crystallized as a white solid upon storage in the freezer (23.3 g, 108 mmol, 96%). No additional purification was required.

R*f* 0.55 (9:1 petroleum ether 40–60/EtOAc);
m.p. = 51–53 °C (petroleum ether 40–60/Et_2O, lit. 48–50 °C);
^{1}H NMR (400 MHz, $CDCl_3$) δ 6.86 – 6.84 (m, 2H), 6.77 – 6.73 (m, 1H), 5.97 (s, 2H), 4.46 (s, 2H) ppm;
^{13}C NMR (100 MHz, $CDCl_3$) δ 148.1, 148.0, 131.7, 122.9, 109.6, 108.5, 101.5, 34.3 ppm;
IR (KBr disc): ν_{max} = 2901, 2784, 1500, 1487, 1444, 1252 cm^{-1};
MS (EI): *m/z* (%): 214 ($M^{+\bullet}$(^{79}Br), 7 %), 135 ($M^{+\bullet}$(^{79}Br) – ^{79}Br, 100 %);
HRMS (EI): calculated for $C_8H_7O_2{}^{79}Br$ $[M]^{+\bullet}$: 213.9629; found 213.9629

Synthesis of endiandric acid A tetrayne precursor

Endiandric tetrayne 16

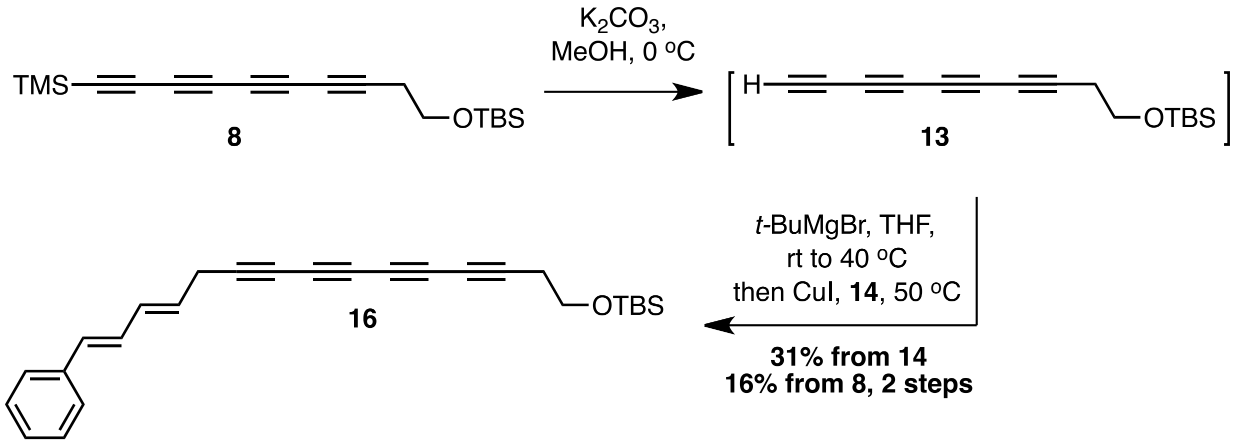

To a 25 mL round-bottomed flask was added TMS tetrayne **8** (0.705 g, 2.15 mmol, 1.0 mol. equiv.) and MeOH (12 mL). The solution was cooled to 0 °C and K_2CO_3 (0.326 g, 2.36 mmol, 1.1 mol. equiv.) was added. The reaction mixture was stirred for a further 10 min under N_2 and then diluted with Et_2O (20 mL) and H_2O (20 mL). One crystal of BHT was added at this time. The aqueous layer was separated and back extracted with additional Et_2O (10 mL). The organic layers were combined, washed with H_2O (10 mL), brine (10 mL), and dried over $MgSO_4$. At this stage the solution containing terminal tetrayne **13** was carefully concentrated at 0 °C to a total volume of ~2.0 mL (*Do not leave unsupervised as terminal tetrayne* ***13*** *decomposes violently and exothermically to intractable products when left neat for periods longer than 1 minute. The utmost care and attention is required. Although this compound is unstable, we found that it could be handled in solution, and was done so reproducibly on gram scale.)* The 'concentrated' solution was then filtered through a short plug of SiO_2 gel (CH_2Cl_2). The filtrate was then carefully concentrated to a total volume of ~2.0 mL, and diluted with $CDCl_3$ (1.0 mL). Durene (0.103 g, 0.767 mmol) was then added, and the amount of intermediate terminal tetrayne **13** quantified by ^1H NMR spectroscopy as 2.04 mmol (95% yield using durene as an internal standard). The remaining solvent was then carefully removed under reduced pressure at 0 °C, and the flask evacuated under high vacuum and back-filled with N_2 (3 × 10 s cycles). Dry THF (9 mL) was then immediately added and the terminal tetrayne **13** solution was subjected to the next step without further purification.

To a dry 3-neck 100 mL round-bottomed flask was added *t*-BuMgBr (0.96 M in THF, 2.76 mL, 2.5 mol. equiv.). The freshly prepared terminal tetrayne **13** solution (2.04 mmol in 9.0 mL dry THF, 2.1 mol. equiv.) was then injected, which resulted in effervescence and a dark orange to deep purple to dark brown colour change. The reaction vessel was heated at 40 °C for a further 30 min (*a small aliquot of the in situ formed tetrayne Grignard reagent was taken and quenched with D_2O to ensure*

that the deprotonation had reached completion) and then cooled to ambient room temperature. The Grignard reagent of terminal tetrayne **13** was then transferred by cannula to a second 3-neck 100 mL round-bottomed flask equipped with an equalising addition funnel. The reaction vessel was then charged with alkyl bromide **14** (1.07 mmol by ^{1}H NMR using durene as an internal standard), recrystallized CuI (61 mg, 0.322 mmol, 0.30 mol. equiv.), and dry THF (4 mL), and the reaction mixture was warmed to 50 °C. The tetrayne Grignard reagent was then added slowly over 20 min and the reaction stirred for a further 30 min once the addition was complete. The reaction was quenched with *sat. aq.* NH_4Cl solution (20 mL) and diluted with Et_2O (20 mL). The aqueous phase was separated and back extracted with Et_2O (20 mL). The organic layers were combined, washed with brine (20 mL), and dried over $MgSO_4$. Concentration under reduced pressure and purification by flash column chromatography (phosphate buffered SiO_2 gel pH 7, 1:0 to 5:1 petroleum ether 40–60/CH_2Cl_2) furnished endiandric tetrayne **16** as an orange oil (0.333 mmol by ^{1}H NMR analysis using durene as an internal standard, 31% from bromide **14**, 16% from **8** over 2 steps).[i]

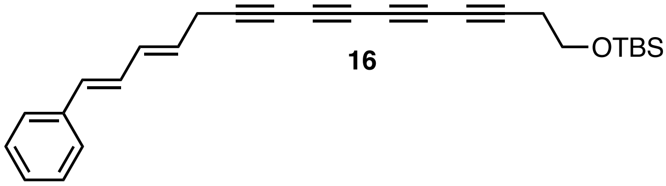

16

R*f* = 0.42 (3:1 petroleum ether 40–60/CH_2Cl_2);

^{1}H NMR (400 MHz, $CDCl_3$) δ 7.39 (d, *J* = 7.5 Hz, 2H), 7.31 (t, *J* = 7.4 Hz, 2H), 7.23 (t, *J* = 7.2 Hz, 1H), 6.75 (dd, *J* = 15.6, 10.4 Hz, 1H), 6.58 (d, *J* = 15.7 Hz, 1H), 6.44 (dd, *J* = 14.9, 10.6 Hz, 1H), 5.70 (dt, *J* = 15.1, 5.7 Hz, 1H), 3.75 (t, *J* = 6.8 Hz, 2H), 3.19 (d, *J* = 5.6 Hz, 2H), 2.52 (t, *J* = 6.7 Hz, 2H), 0.90 (s, 9H), 0.076 (s, 6H) ppm;

^{13}C NMR (100 MHz, $CDCl_3$) δ 137.2 (C_q), 133.2 (CH), 132.7 (CH), 128.8 (CH), 127.9 (CH), 127.8 (CH), 126.5 (CH), 125.5 (CH), 78.0 (C_q), 76.7 (C_q), 67.8 (C_q), 66.8 (C_q), 61.9 (C_q), 61.8 (C_q), 61.3 (C_q), 61.2 (CH_2), 60.8 (C_q), 26.0 (CH_3), 24.1 (CH_2), 23.0 (CH_2), 18.4 (C_q), –5.2 (CH_3) ppm;

IR (thin film): ν_{max} = 2955, 2857, 2227, 1257, 1107 cm^{-1};

MS (EI): *m/z* (%): 398 ($M^{+\bullet}$, 10 %), 341 ($M^{+\bullet}$– ^{t}Bu, 75 %);

HRMS (EI): calculated for $C_{27}H_{30}OSi$ $[M]^{+\bullet}$: 398.2066; found 398.2065.

[i] Although 0.228 g of endiandric tetrayne **16** material was isolated by silica gel column chromatography, subsequent ^{1}H NMR analysis and calibration against an internal standard found the material to be 58% pure by mass, which equated to 0.333 mmol of **16** at 31% yield from bromide **14**, or 16% over two steps from TMS tetrayne **8**. This material was used without further purification.

*Total synthesis of (±)-endiandric acid A (**1**)*

(±)-Alcohol 23

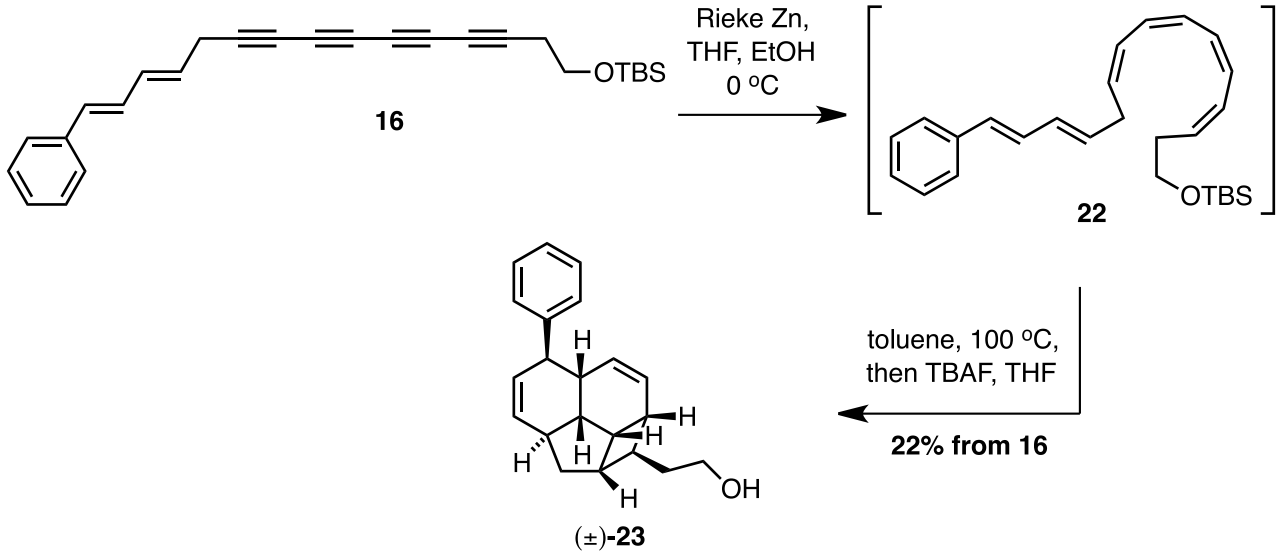

Rieke Zinc preparation and tetrayne reduction

A 3-neck 250 mL round-bottomed flask, equipped with a large magnetic stir bar and a crushed glass pipette tip, was flame-dried under high vacuum, and then cooled under dry Ar. The reaction vessel was charged with naphthalene (0.564 g, 4.40 mmol, 7.6 mol. equiv.) and dry THF (6.0 mL). Finely sliced Li metal (0.610 g, 88.0 mmol, 340 mol. equiv.) was added and the mixture was stirred vigorously under Ar until the deep green lithium naphthalenide complex was observed. The mixture was stirred for an additional 15 min, then cooled to 0 °C, and anhydrous $ZnBr_2$ (1.50 M in dry THF, 29.3 mL, 44.0 mmol, 170 mol. equiv.) was added slowly over 10 min. A fine black zinc suspension developed over the course of addition. The Rieke zinc solution was stirred for a further 1 h at ambient room temperature prior to use.

The reaction vessel containing Rieke zinc (approx. 170 mol. equiv.) was cooled to 0 °C and endiandric tetrayne **16** (0.195 g, 0.258 mmol by ^{1}H NMR analysis using durene as an internal standard, 1.0 mol. equiv.) dissolved in dry THF (4.0 mL) and dry EtOH (7.0 mL) were added simultaneously. The resulting mixture was stirred at 0 °C for 15 h under Ar in the dark until complete reduction to the tetraene had occurred *(GCMS, ^{1}H and ^{13}C NMR monitoring)*. The reaction was then cautiously quenched with *sat. aq*. NH_4Cl solution (50 mL) at 0 °C under Ar, and left to stir for 1 h until all remaining lithium metal had reacted *(note: light exposure was minimised during work up)*. The resulting mixture was diluted with Et_2O (50 mL). The aqueous layer was separated and back extracted with additional Et_2O (50 mL). The organic layers were combined, washed with brine (50 mL) and dried over $MgSO_4$. Removal of naphthalene by filtration (SiO_2, trace BHT in solvent, petroleum ether 40–60 to 3:1 petroleum ether 40–60/CH_2Cl_2) afforded

(*Z,Z,Z,Z*)-tetraene **22** as the major diastereomer along with inseparable minor diastereomers (0.135 g crude mass). Extensive analytical HPLC studies failed to establish an accurate diastereomeric ratio of the (*Z,Z,Z,Z*)-tetraene **22** to minor diastereomers. To avoid additional undesired (*Z/E*)-isomerisation, the tetraene material was immediately subjected to the next step.

One-pot 8π-6π electrocyclization – intramolecular Diels–Alder – deprotection

The tetraene mixture (0.135 g crude mass) containing (*Z,Z,Z,Z*)-tetraene **22** was dissolved in dry toluene (7.0 mL) and heated at 100 °C in the dark for 20 h under N_2. After this time the reaction vessel was cooled to ambient room temperature and toluene was removed by concentration under reduced pressure. Dry THF (3.0 mL) and TBAF (1.0 M in THF, 0.70 mL, approx. 2.0 mol. equiv.) were then added and the reaction stirred for 45 min. The reaction was quenched with *sat. aq.* NH_4Cl solution (20 mL) and diluted with Et_2O (20 mL). The aqueous phase was separated and back extracted with Et_2O (20 mL). The organic layers were combined, washed with brine (30 mL), and dried over $MgSO_4$. Concentration under reduced pressure and purification by flash column chromatography (10% $AgNO_3$ – SiO_2,[7] 1:1 to 1:9 petroleum ether 40–60/Et_2O) afforded (±)-alcohol **23** as a yellow oil (16.7 mg, 0.0571 mmol, 22% from **16**, 2 steps).

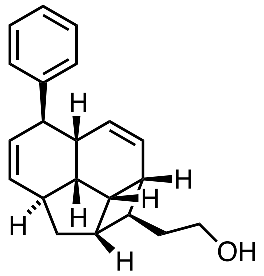

(±)-**23**

R*f* = 0.51 (1:1 petroleum ether 40–60/Et_2O);

^{1}H NMR (400 MHz, $CDCl_3$) δ 7.34 – 7.24 (m, 4H), 7.20 (tm, *J* = 7.2 Hz, 1H), 6.21 (dt, *J* = 9.6, 2.3 Hz, 1H), 5.70 – 5.60 (m, 3H), 3.63 (t, *J* = 6.7 Hz, 2H), 3.30 – 3.26 (m, 1H), 2.73 – 2.64 (m, 1H), 2.60 (q, *J* = 7.4 Hz, 1H), 2.53 – 2.48 (m, 1H), 2.35 (q, *J* = 5.7 Hz, 1H), 2.33 – 2.25 (m, 1H), 1.80 (qm, *J* = 7.4 Hz, 3H), 1.70 – 1.60 (m, 2H), 1.39 (td, *J* = 12.4, 5.8 Hz, 1H) ppm;

^{13}C NMR (100 MHz, $CDCl_3$) δ 147.8 (C_q), 132.0 (CH), 131.5 (CH), 130.6 (CH), 128.6 (CH), 128.2 (CH), 127.6 (CH), 126.2 (CH), 61.1 (CH_2), 50.5 (CH), 42.5 (CH), 41.5 (CH), 41.4 (CH), 40.2 (CH_2), 39.4 (CH), 37.0 (CH), 35.1 (CH), 35.1 (CH_2), 33.6 (CH) ppm;

IR (thin film): ν_{max} = 3368, 2924, 1671, 1076 cm^{-1};

MS (EI): *m/z* (%): 292 ($M^{+\bullet}$, 25 %), 214 ($M^{+\bullet}$– C_6H_6, 60 %);

HRMS (EI): calculated for $C_{21}H_{24}O$ $[M]^{+\bullet}$: 292.1827; found 292.1830.

(±)-Endiandric acid A (1)

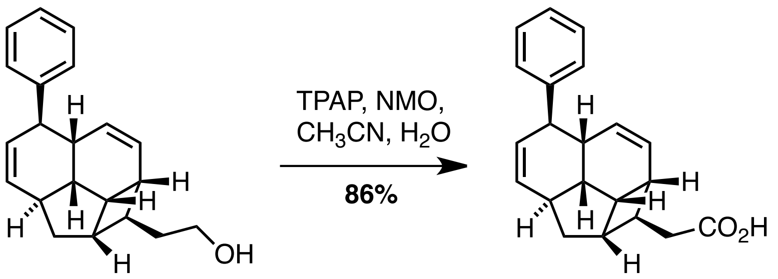

(±)-**23** (±)-**endiandric acid A (1)**

To a 10 mL round-bottomed flask containing CH_3CN:H_2O (0.62 mL, 95:5) was added (±)-alcohol **23** (19.5 mg, 0.0667 mmol, 1.0 mol. equiv.) under N_2. NMO (0.195 g, 1.67 mmol, 25 mol. equiv.) and TPAP (5.9 mg, 0.017 mmol, 0.25 mol. equiv.) were then added, and the reaction stirred at ambient room temperature for 15 min. The reaction was then quenched with 1 M HCl (4 mL) and diluted with EtOAc (4 mL). The aqueous phase was separated and back extracted with additional EtOAc (2 × 4 mL). The organic layers were combined, washed with 1 M HCl (3 × 5 mL), then brine (5 mL), and dried over $MgSO_4$. Concentration under reduced pressure furnished (±)-endiandric acid A (**1**) as a pale-orange paste (17.5 mg, 0.0571 mmol, 86%). The ^{1}H and ^{13}C NMR of synthetic (±)-endiandric acid A (**1**) was identical in all respects to that of a natural (±)-endiandric acid A sample kindly provided by Prof David St Clair Black.

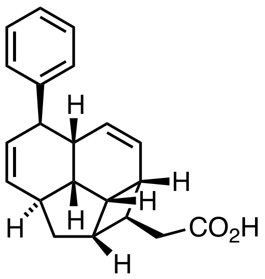

(±)-**endiandric acid A (1)**

R*f* = 0.41 (1:1 petroleum ether 40–60/Et_2O);

^{1}H NMR (400 MHz, $CDCl_3$) δ 7.33 – 7.18 (m, 5H), 6.21 (dt, *J* = 9.7, 2.0 Hz, 1H), 5.70 – 5.64 (m, 3H), 3.31 – 3.28 (m, 1H), 2.75 – 2.47 (m, 5H), 2.45 – 2.33 (m, 2H), 2.04 – 1.93 (m, 1H), 1.85 – 1.77 (m, 1H), 1.75 (dd, *J* = 12.0, 5.5 Hz, 1H), 1.39 (ddd, *J* = 12.8, 5.8, 5.7 Hz, 1H) ppm;

^{13}C NMR (100 MHz, $CDCl_3$) δ 178.6 (C_q), 147.8 (C_q), 131.8 (2CH coincident), 130.6 (CH), 128.6 (CH), 127.8 (CH), 127.6 (CH), 126.2 (CH), 50.5 (CH), 41.5 (CH), 41.5 (CH), 41.4 (CH), 40.9 (CH_2), 39.4 (CH), 36.8 (CH), 35.0 (CH), 34.9 (CH_2), 33.5 (CH) ppm;

IR (thin film): ν_{max} = 3154, 3023, 2935, 1706 cm^{-1};

MS (ESI): 329 ([*M* +Na]$^+$, 100 %);

HRMS (ESI): calculated for $C_{21}H_{22}O_2{}^{23}Na$ [*M* + Na]$^+$: 329.1517; found 329.1519.

Synthesis of kingianic acid E tetrayne precursor

Kingianic tetrayne 17

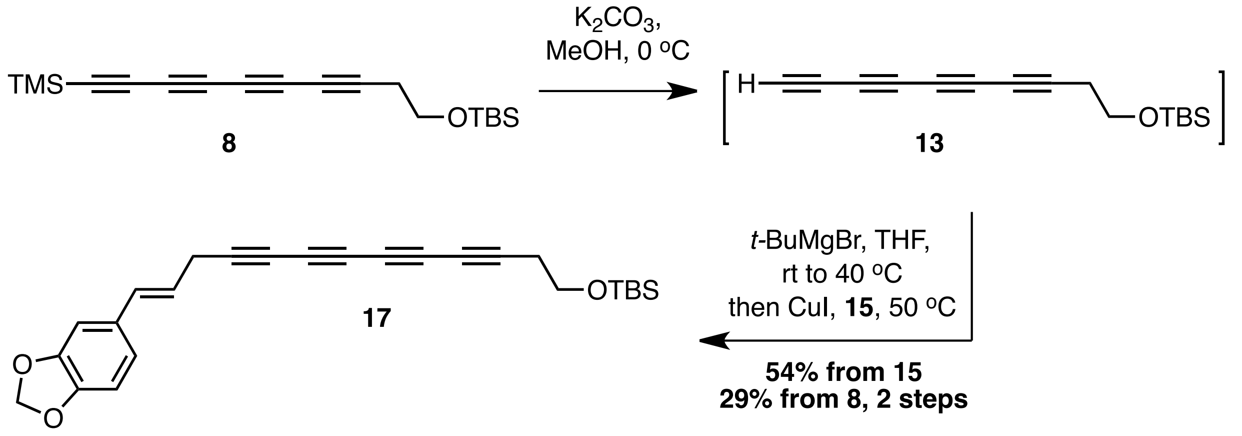

To a 50 mL round-bottomed flask was added TMS tetrayne **8** (1.12 g, 3.40 mmol, 1.0 mol. equiv.) and MeOH (19 mL). The solution was cooled to 0 °C and K_2CO_3 (0.516 g, 3.74 mmol, 1.1 mol. equiv.) was added. The reaction mixture was stirred for a further 10 min under N_2 and then diluted with Et_2O (30 mL) and H_2O (30 mL). One crystal of BHT was added at this time. The aqueous layer was separated and back extracted with additional Et_2O (30 mL). The organic layers were combined, washed with H_2O (30 mL), brine (30 mL), and dried over $MgSO_4$. At this stage the solution containing terminal tetrayne **13** was carefully concentrated at 0 °C to a total volume of ~4.0 mL (*Do not leave unsupervised as terminal tetrayne* ***13*** *decomposes violently and exothermically to intractable products when left neat for periods longer than 1 minute. The utmost care and attention is required. Although this compound is unstable, we found that it could be handled in solution, and was done so reproducibly on gram scale.)* The 'concentrated' solution was then filtered through a short plug of SiO_2 gel (CH_2Cl_2). The filtrate was then carefully concentrated to a total volume of ~4.0 mL, and diluted with $CDCl_3$ (1.0 mL). Durene (0.148 g, 1.11 mmol) was then added, and the amount of intermediate terminal tetrayne **13** quantified by ^1H NMR spectroscopy as 2.99 mmol (88% using durene as an internal standard). The remaining solvent was then carefully removed under reduced pressure at 0 °C, and the flask evacuated under high vacuum and back-filled with N_2 (3 × 10 s cycles). Dry THF (15 mL) was then immediately added and the terminal tetrayne **13** solution was subjected to the next step without further purification.

To a dry 3-neck 100 mL round-bottomed flask was added *t*-BuMgBr (0.96 M in THF, 4.04 mL, 2.1 mol. equiv.). The freshly prepared terminal tetrayne **13** solution (2.99 mmol in 15.0 mL dry THF, 1.65 mol. equiv.) was then injected, which resulted in effervescence and a dark orange to deep purple to dark brown colour change. The reaction vessel was heated at 40 °C for a further 30 min (*a small aliquot of the in situ formed tetrayne Grignard reagent was taken and quenched with D_2O to*

ensure that the deprotonation had reached completion) and then cooled to ambient room temperature. The Grignard reagent of terminal tetrayne **13** was then transferred by cannula to a second 3-neck 100 mL round-bottomed flask equipped with an equalising addition funnel. The reaction vessel was then charged with alkyl bromide **15** (1.80 mmol by ^{1}H NMR analysis using durene as an internal standard), recrystallized CuI (0.102 g, 0.540 mmol, 0.30 mol. equiv.), and dry THF (6 mL), and the resulting solution was warmed to 50 °C. The tetrayne Grignard reagent was then added slowly over 20 min and the reaction stirred for a further 30 min once the addition was complete. The reaction was quenched with *sat. aq.* NH_4Cl solution (30 mL) and diluted with Et_2O (30 mL). The aqueous phase was separated and back extracted with Et_2O (30 mL). The organic layers were combined, washed with brine (20 mL), and dried over $MgSO_4$. Concentration under reduced pressure and purification by flash column chromatography (phosphate buffered SiO_2 gel pH 7, 1:0 to 3:1 petroleum ether 40–60/CH_2Cl_2) furnished kingianic tetrayne **17** as a dark orange oil (0.972 mmol by ^{1}H NMR analysis using durene as an internal standard, 54% from bromide **15**, 29% from **8** over 2 steps).[ii]

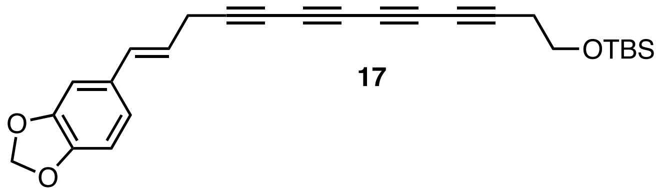

17

R$_f$ = 0.23 (4:1 petroleum ether 40–60/CH_2Cl_2);

^{1}H NMR (400 MHz, $CDCl_3$) δ 6.88 (s, 1H), 6.82 – 6.73 (m, 2H), 6.54 (d, J = 15.7 Hz, 1H), 5.95 (s, 2H), 5.90 (dt, J = 15.7, 5.7 Hz, 1H), 3.75 (t, J = 6.8 Hz, 2H), 3.21 (d, J = 7.3 Hz, 2H), 2.53 (t, J = 6.8 Hz, 2H), 0.90 (s, 9H), 0.078 (s, 6H) ppm;

^{13}C NMR (100 MHz, $CDCl_3$) δ 148.2 (C_q), 147.4 (C_q), 132.3 (CH), 131.2 (C_q), 121.1 (CH), 120.0 (CH), 108.4 (CH), 105.8 (CH), 101.2 (CH_2), 78.0 (C_q), 76.9 (C_q), 67.8 (C_q), 66.8 (C_q), 61.9 (C_q), 61.8 (C_q), 61.4 (C_q), 61.2 (CH_2), 60.8 (C_q), 26.0 (CH_3), 24.1 (CH_2), 23.0 (CH_2), 18.4 (C_q), –5.2 (CH_3) ppm;

IR (thin film): ν_{max} = 2954, 2929, 2856, 2227, 1503, 1490, 1251, 1106 cm^{-1};

MS (EI): *m/z* (%): 416 ($M^{+\bullet}$, 10 %), 359 ($M^{+\bullet}$– ^{t}Bu, 50 %);

HRMS (EI): calculated for $C_{26}H_{28}O_3Si$ $[M]^{+\bullet}$: 416.1808; found 416.1808.

[ii] Although 0.539 g of kingianic tetrayne **17** material was isolated by silica gel column chromatography, subsequent ^{1}H NMR analysis and calibration against an internal standard found the material to be 75% pure by mass, which equated to 0.972 mmol of **17** at 54% yield from bromide **15**, or 29% over two steps from TMS tetrayne **8**. This material was used without further purification.

Total synthesis of (±)-kingianic acid E (3)

(±)-Alcohol 25

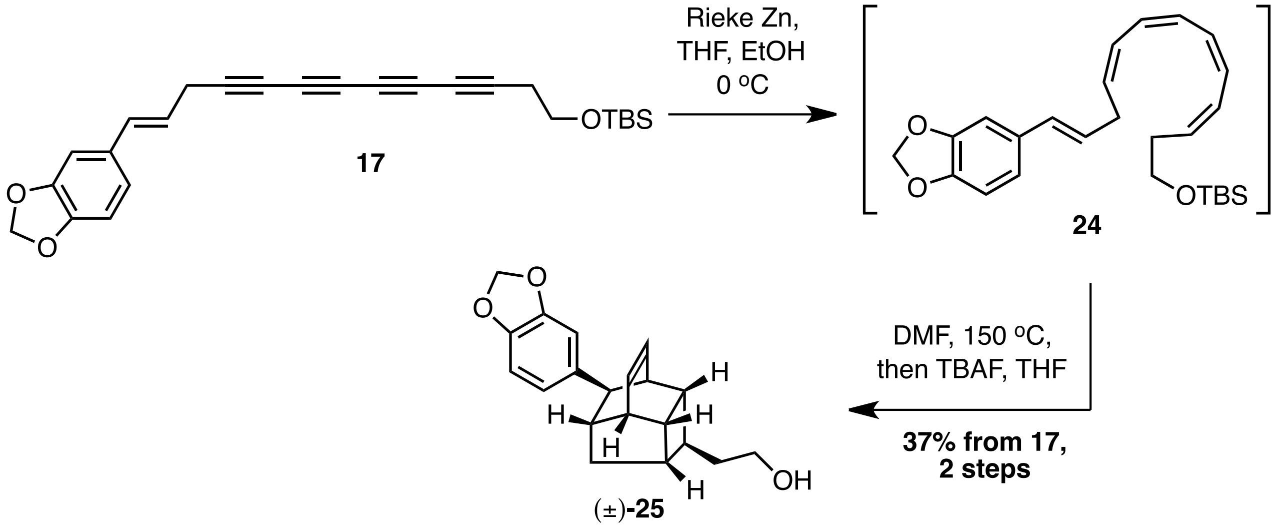

Rieke Zinc preparation and tetrayne reduction

A 3-neck 250 mL round-bottomed flask, equipped with a large magnetic stir bar and a crushed glass pipette tip, was flame-dried under high vacuum, and then cooled under dry Ar. The reaction vessel was charged with naphthalene (0.553 g, 4.32 mmol, 13.1 mol. equiv.) and dry THF (6.0 mL). Finely sliced Li metal (0.600 g, 86.4 mmol, 263 mol. equiv.) was added and the mixture was stirred vigorously under Ar until the deep green lithium naphthalenide complex was observed. The mixture was stirred for an additional 15 min, then cooled to 0 °C, and anhydrous $ZnBr_2$ (1.50 M in dry THF, 28.8 mL, 43.2 mmol, 131 mol. equiv.) was added slowly over 10 min. A fine black zinc suspension developed over the course of addition. The Rieke zinc solution was stirred for a further 1 h at ambient temperature prior to use.

The reaction vessel containing Rieke zinc (approx. 131 mol. equiv.) was cooled to 0 °C and unsymmetrical tetrayne **17** (0.200 g, 0.329 mmol by ^{1}H NMR analysis using durene as an internal standard, 1.0 mol. equiv.) dissolved in dry THF (3.0 mL) and dry EtOH (7.0 mL) were added simultaneously. The resulting mixture was stirred at 0 °C for 15 h under Ar in the dark until complete reduction to the tetraene had occurred *(GCMS, ^{1}H and ^{13}C NMR monitoring)*. The reaction was then cautiously quenched with *sat. aq*. NH_4Cl solution (50 mL) at 0 °C under Ar, and left to stir for 1 h until all remaining lithium metal had reacted *(note: light exposure was minimised during work up)*. The resulting mixture was diluted with Et_2O (50 mL). The aqueous layer was separated and back extracted with additional Et_2O (50 mL). The organic layers were combined, washed with brine (50 mL) and dried over $MgSO_4$. Removal of naphthalene by filtration (SiO_2, trace BHT in solvent, petroleum ether 40–60 to 3:1 petroleum ether 40–60/CH_2Cl_2) afforded (*Z,Z,Z,Z*)-tetraene **24** as the major diastereomer along with inseparable minor diastereomers (0.143

g crude mass). Extensive analytical HPLC studies failed to establish an accurate diastereomeric ratio of the (*Z,Z,Z,Z*)-tetraene **24** to minor diastereomers. To avoid additional undesired *Z/E*-isomerisation, the tetraene material was immediately subjected to the next step.

One-pot 8π–6π electrocyclization – intramolecular Diels–Alder – deprotection

The tetraene mixture (0.143 g crude mass) containing (*Z,Z,Z,Z*)-tetraene **24** was dissolved in dry DMF (3.0 mL) and heated at 150 °C in the dark for 20 h under N_2. After this time the reaction mixture was cooled to ambient room temperature and TBAF (1.0 M in THF, 0.70 mL, approx. 2.0 mol. equiv.) was added and the reaction stirred for 1 h. The reaction was quenched with *sat. aq.* NH_4Cl solution (20 mL) and diluted with Et_2O (20 mL). The aqueous phase was separated and back extracted with Et_2O (20 mL). The organic layers were combined, washed with brine (30 mL), and dried over $MgSO_4$. Concentration under reduced pressure and purification by flash column chromatography (10% $AgNO_3$ – SiO_2,[7] 4:1 to 2:1 petroleum ether 40–60/EtOAc) afforded alcohol **(±)-25** as a yellow-orange oil (37.4 mg, 0.120 mmol, 37% from **17**, 2 steps).

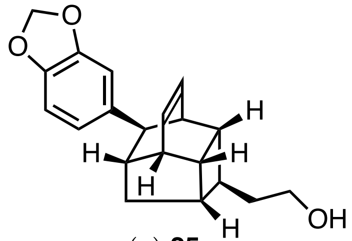

(±)-**25**

R_f = 0.20 (2:1 petroleum ether 40–60/Et_2O);

^{1}H NMR (400 MHz, $CDCl_3$) δ 6.67 (d, J = 8.0 Hz, 1H), 6.60 (s, 1H), 6.54 (d, J = 8.0 Hz, 1H), 6.29 (t, J = 7.3 Hz, 1H), 5.93 (t, J = 6.8 Hz, 1H), 5.89 (s, 2H), 3.70 (t, J = 6.8 Hz, 2H), 3.24 (br s, 1H), 2.77 (q, J = 5.3 Hz, 1H), 2.70 – 2.62 (m, 1H), 2.49 – 2.41 (m, 1H), 2.35 – 2.26 (m, 2H), 2.11 (t, J = 7.6 Hz, 1H), 1.98 – 1.79 (m, 3H), 1.73 – 1.66 (m, 2H) ppm;

^{13}C NMR (100 MHz, $CDCl_3$) δ 147.0 (C_q), 145.3 (C_q), 140.6 (C_q), 132.4 (CH), 130.6 (CH), 121.7 (CH), 109.7 (CH), 107.5 (CH), 100.8 (CH_2), 61.3 (CH_2), 48.1 (CH), 43.4 (CH), 42.6 (CH), 40.8 (CH), 40.6 (CH), 40.2 (CH), 40.0 (CH), 39.8 (CH_2), 39.6 (CH_2), 35.5 (CH) ppm;

IR (thin film): ν_{max} = 3369, 2921, 1502, 1489, 1039 cm^{-1};

MS (EI): *m/z* (%): 310 ($M^{+\bullet}$, 30 %), 207 (100 %);

HRMS (EI): calculated for $C_{20}H_{22}O_3$ $[M]^{+\bullet}$: 310.1569; found 310.1567.

(±)-Kingianic acid E (3)

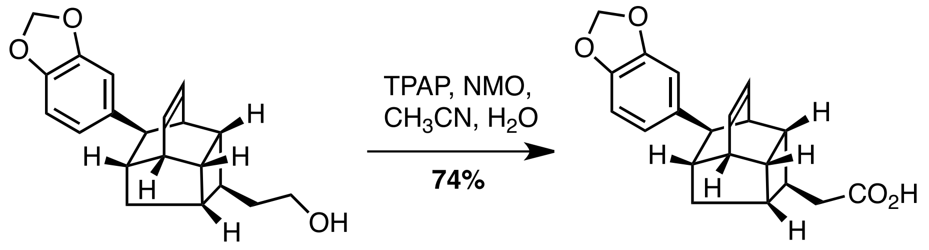

To a 10 mL round-bottomed flask containing CH_3CN:H_2O (1.50 mL, 95:5) was added alcohol **(±)-25** (37.4 mg, 0.120 mmol, 1.0 mol. equiv.) under N_2. NMO (0.351 g, 3.00 mmol, 25 mol. equiv.) and TPAP (11.0 mg, 0.030 mmol, 0.25 mol. equiv.) were then added, and the reaction stirred at ambient room temperature for 15 min. The reaction was then quenched with 1 M HCl (4 mL) and diluted with EtOAc (4 mL). The aqueous phase was separated and back extracted with additional EtOAc (2 × 4 mL). The organic layers were combined, washed with 1 M HCl (3 × 5 mL), then brine (5 mL), and dried over $MgSO_4$. Concentration under reduced pressure furnished (±)-kingianic acid E (**3**) as an orange oil (29.0 mg, 0.0894 mmol, 74%). The ^1H and ^{13}C NMR of synthetic (±)-kingianic acid E (**3**) matched that reported by Litaudon and co-workers.[8]

R_f = 0.70 (Et_2O);

^{1}H NMR (600 MHz, $CDCl_3$) δ 6.68 (d, J = 7.9 Hz, 1H), 6.61 (s, 1H), 6.55 (d, J = 8.0 Hz, 1H), 6.29 (t, J = 7.3 Hz, 1H), 5.93 (t, J = 7.0 Hz, 1H), 5.89 (s, 2H), 3.25 (br s, 1H), 2.78 (br q, J = 4.8 Hz, 1H), 2.74 – 2.70 (m, 1H), 2.69 – 2.60 (m, 2H), 2.50 – 2.43 (m, 2H), 2.40 – 2.35 (m, 1H), 2.34 – 2.28 (m, 1H), 1.98 – 1.90 (m, 1H), 1.81 – 1.72 (m, 2H) ppm;

^{13}C NMR (200 MHz, $CDCl_3$) δ 178.7 (C_q), 147.1 (C_q), 145.3 (C_q), 140.3 (C_q), 132.4 (CH), 130.6 (CH), 121.7 (CH), 109.6 (CH), 107.5 (CH), 100.8 (CH_2), 48.0 (CH), 43.3 (CH), 42.5 (CH), 40.7 (CH_2), 40.5 (CH), 40.4 (CH), 40.1 (CH), 39.7 (CH), 39.5 (CH_2), 35.4 (CH) ppm;

IR (thin film): ν_{max} = 2929, 1703, 1503, 1489, 1235, 1039 cm^{-1};

MS (ESI): 347 ([M +Na]$^+$, 68 %), 379 (100 %);

HRMS (ESI): calculated for $C_{20}H_{20}O_4{}^{23}Na$ [M + Na]$^+$: 347.1259; found 347.1259.

NMR comparison tables for (±)-Kingianic acid E (3)

*^{1}H NMR of (±)-kingianic acid E (**3**)*

	Our Synthetic Sample (600 MHz, $CDCl_3$) ppm	***Litaudon's natural sample[8] (600 MHz, $CDCl_3$) ppm***
1	6.68 (d, *J* = 7.9 Hz, 1H)	6.67 (d, *J* = 8.0 Hz, 1H)
2	6.61 (s, 1H)	6.61 (s, 1H)
3	6.55 (d, *J* = 8.0 Hz, 1H)	6.54 (d, *J* = 8.0 Hz, 1H)
4	6.29 (t, *J* = 7.3 Hz, 1H)	6.29 (t, *J* = 7.3 Hz, 1H)
5	5.93 (t, *J* = 7.0 Hz, 1H)	5.93 (t, *J* = 7.0 Hz, 1H)
6	5.89 (s, 2H)	5.89 (s, 2H)
7	3.25 (br s, 1H)	3.25 (d, *J* = 2.5 Hz, 1H)
8	2.78 (br q, *J* = 4.8 Hz, 1H)	2.76 (dd, *J* = 10.8, 5.1 Hz, 1H)
9	2.74 – 2.70 (m, 1H)	2.72 (m, 1H)
10	2.69 – 2.60 (m, 2H)	2.64 (m, 2H)
11	2.50 – 2.43 (m, 2H)	2.62 (m, 1H)
12		2.46 (m, 1H)
13		2.44 (m, 1H)
14	2.40 – 2.35 (m, 1H)	2.37 (t, *J* = 6.6 Hz, 1H)
15	2.34 – 2.28 (m, 1H)	2.31 (t, *J* = 4.6 Hz, 1H)
16	1.98 – 1.90 (m, 1H)	1.94 (m, 1H)
17	1.81 – 1.72 (m, 2H)	1.74 (d, *J* = 12.6 Hz, 1H)

*^{13}C NMR of (±)-kingianic acid E (**3**)*

	Our Synthetic Sample (200 MHz, $CDCl_3$) ppm	***Litaudon's natural sample[8] (150 MHz, $CDCl_3$) ppm[iii]***
1	178.7	176.9
2	147.1	146.9
3	145.3	145.2
4	140.3	140.2
5	132.4	132.3
6	130.6	130.5
7	121.7	121.6
8	109.6	109.5
9	107.5	107.4
10	100.8	100.7
11	48.0	47.9
12	43.3	43.1
13	42.5	42.4
14	40.7	40.4
15	40.5	40.3
16	40.4	40.2
17	40.1	39.9
18	39.7	39.6
19	39.5	39.3
20	35.4	35.3

iii Litaudon *et al.* referenced $CDCl_3$ to 77.01 ppm.

Formal synthesis of kingianins A, D, and F

Kingianin tetrayne 18

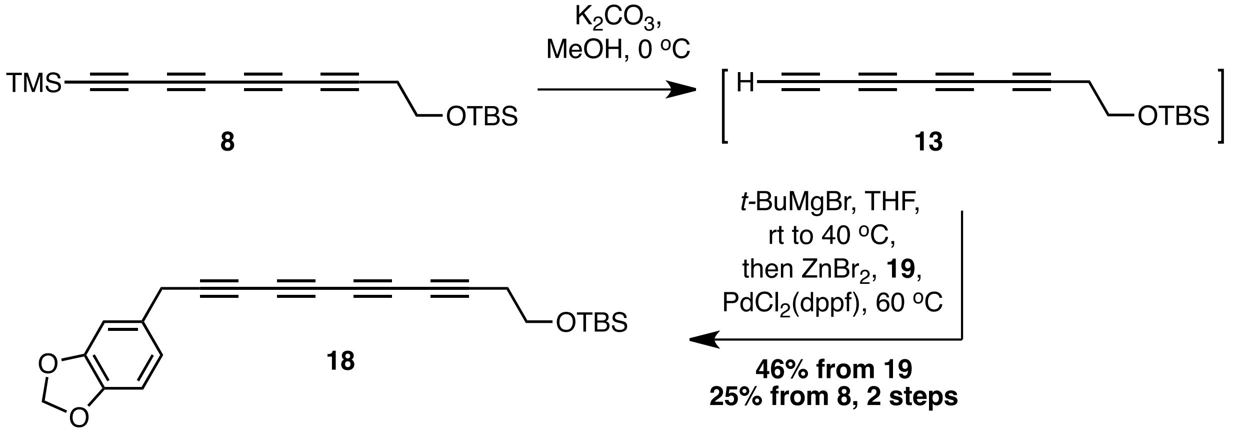

To a 25 mL round-bottomed flask was added TMS tetrayne **8** (0.200 g, 0.609 mmol, 1.0 mol. equiv.) and MeOH (3.0 mL). The solution was cooled to 0 °C and K_2CO_3 (0.093g, 0.670 mmol, 1.1 mol. equiv.) was added. The reaction mixture was stirred for a further 10 min under N_2 and then diluted with Et_2O (5 mL) and H_2O (5 mL). One crystal of BHT was added at this time. The aqueous layer was separated and back extracted with additional Et_2O (10 mL). The organic layers were combined, washed with H_2O (10 mL), brine (10 mL), and dried over $MgSO_4$. At this stage the solution containing terminal tetrayne **13** was carefully concentrated at 0 °C to a total volume of ~2 mL (*Do not leave unsupervised as terminal tetrayne **13** decomposes violently and exothermically to intractable products when left neat for periods longer than 1 minute. The utmost care and attention is required. Although this compound is unstable, we found that it could be handled in solution, and was done so reproducibly on gram scale.)* The 'concentrated' solution was then filtered through a short plug of SiO_2 gel (CH_2Cl_2). The filtrate was then carefully concentrated to a total volume of ~2 mL, and diluted with $CDCl_3$ (1.0 mL). Durene (0.104 g, 0.773 mmol) was then added, and the amount of intermediate terminal tetrayne **13** quantified by ^{1}H NMR spectroscopy as 0.554 mmol (91% using durene as an internal standard). The remaining solvent was then carefully removed under reduced pressure at 0 °C, and the flask evacuated under high vacuum and back-filled with N_2 (3 × 10 s cycles). Dry THF (3.0 mL) was then immediately added and the terminal tetrayne **13** solution was subjected to the next step without further purification.

To a dry 3-neck 50 mL round-bottomed flask equipped with a reflux condenser was added *t*-BuMgBr (0.96 M in THF, 0.75 mL, 2.2 mol. equiv.). The freshly prepared terminal tetrayne **13** solution (0.554 mmol in 3.0 mL dry THF, 1.7 mol. equiv.) was then injected, which resulted in effervescence and a dark orange to deep purple to dark brown colour change. The reaction vessel was heated at 40 °C for a further 30 min (*a small aliquot of the in situ formed tetrayne Grignard*

reagent was taken and quenched with D_2O to ensure that the deprotonation/metalation had reached completion) and then cooled to 0 °C. Dry $ZnBr_2$ (1.50 M in THF, 0.48 ml, 0.721 mmol, 2.2 mol. equiv.) was then added and the reaction vessel was warmed to ambient room temperature and stirred for 30 min. Bromide **19** (0.070 g, 0.326 mmol, 1.0 mol. equiv.) and $PdCl_2$(dppf) (7.2 mg, 0.0098 mmol, 3.0 mol%) were then added simultaneously and the reaction vessel was placed in a pre-heated 60 °C oil bath and stirred for 17 h. The reaction was quenched with *sat. aq.* NH_4Cl solution (10 mL) and diluted with Et_2O (10 mL). The aqueous phase was separated and back extracted with Et_2O (10 mL). The organic layers were combined, washed with brine (10 mL), and dried over $MgSO_4$. Concentration under reduced pressure and purification by flash column chromatography (phosphate buffered SiO_2 gel pH 7, 1:0 to 2:1 petroleum ether 40–60/CH_2Cl_2) furnished kingianin tetrayne **18** (0.059 g, 0.150 mmol, 46% from bromide **19**, 25% over 2 steps from **8**). The synthesis of unsymmetrical tetrayne **18** constitutes a formal total synthesis of kingianins A (**2**), D (**20**), and F (**21**).[9]

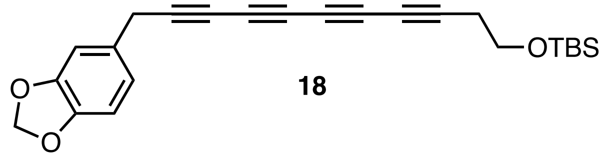

18

R$_f$ = 0.30 (3:1 petroleum ether 40–60/CH_2Cl_2);

^{1}H NMR (400 MHz, $CDCl_3$) δ 6.81 – 6.70 (m, 3H), 5.95 (s, 2H), 3.75 (t, J = 6.8 Hz, 2H), 3.62 (s, 2H), 2.52 (t, J = 6.8 Hz, 2H), 0.89 (s, 9H), 0.07 (s, 6H) ppm;

^{13}C NMR (100 MHz, $(CD_3)_2CO$) δ 149.0, 147.7, 129.2, 122.0, 109.3, 109.1, 102.1, 80.6, 79.9, 67.1, 66.4, 62.7, 61.9, 61.7, 61.6, 60.6, 26.2, 25.5, 24.3, 18.8, −5.2 ppm;

IR (thin film): ν_{max} = 2939, 2928, 2227, 1502, 1488, 1248 cm^{-1};

MS (EI): *m/z* (%): 390 ($M^{+\bullet}$, 10 %), 375 ($M^{+\bullet}$– CH_3, 4 %), 333 ($M^{+\bullet}$ – C_4H_9, 100 %);

HRMS (EI): calculated for $C_{24}H_{26}O_3Si$ $[M]^{+\bullet}$: 390.1651; found 390.1650.

Isolation of IMDA precursor (±)-*bicyclo[4.2.0]octadiene* ***26***

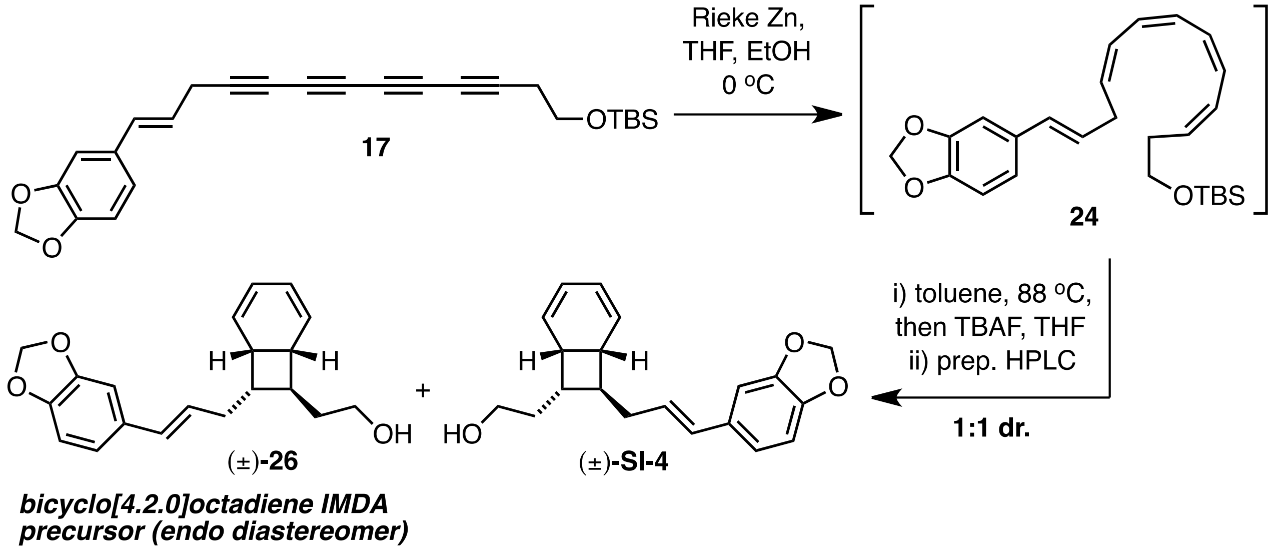

Initial thermal studies of (*Z,Z,Z,Z*)-tetraene **24** (prepared as described above) at 100 °C led to product mixtures with the IMDA adduct **25** as the minor component. Upon further examination, it was found that the thermal pericyclic sequence could be effectively stopped after the 8π–6π electrocyclization by restricting the heating of (*Z,Z,Z,Z*)-tetraene **24** to 88 °C in toluene (0.07 M) over a period of 21 h. Upon TBAF deprotection, conducted in the same manner as per the synthesis of (±)-alcohol **25**, analytically pure samples of (±)-bicyclo[4.2.0]octadienes **26** and **SI-4** could be obtained by flash column chromatography (SiO_2, 5:1 to 3:1 petroleum ether 40–60/EtOAc) followed by preparative reverse phase HPLC (Gemini C_{18} 5μ 150 × 21.20 mm column, 68:32 MeOH/H_2O, 21.0 ml/min flow rate; (±)-bicyclo[4.2.0]octadiene (**SI-4**) t_R = 23.2 min; (±)-bicyclo[4.2.0]octadiene (**26**) t_R = 25.0 min).

(±)-Bicyclo[4.2.0]octadiene 26

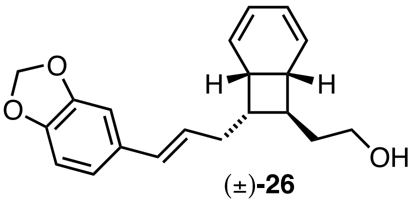

Isolated as a pale yellow oil.

R*f* = 0.19 (2:1 petroleum ether 40–60/Et_2O);

^{1}H NMR (400 MHz, CD_2Cl_2) δ 6.89 (s, 1H), 6.80 – 6.67 (m, 2H), 6.32 (d, *J* = 15.8 Hz, 1H), 6.03 (dt, *J* = 15.8, 6.9 Hz, 1H), 5.93 (s, 2H), 5.91 – 5.85 (m, 1H), 5.71 – 5.55 (m, 3H), 3.52 (br t, *J* = 5.9 Hz, 2H), 3.20 (br t, *J* = 8.8 Hz, 1H), 2.66 – 2.51 (m, 2H), 2.51 – 2.35 (m, 3H), 1.78 – 1.57 (m, 2H) ppm;

^{13}**C NMR** (100 MHz, CD_2Cl_2) δ 148.4 (C_q), 147.1 (C_q), 132.8 (C_q), 130.2 (CH), 128.2 (CH), 127.3 (CH), 127.0 (CH), 124.5 (CH), 121.9 (CH), 120.6 (CH), 108.5 (CH), 105.5 (CH), 101.6 (CH_2), 61.8 (CH_2), 51.1 (CH), 49.2 (CH), 39.5 (CH_2), 37.3 (CH), 35.0 (CH), 34.4 (CH_2) ppm;

IR (thin film): ν_{max} = 3375, 2927, 1793, 1487, 1096 cm^{-1};

MS (EI): *m/z* (%): 310 ($M^{+\bullet}$, 15 %), 232 ($M^{+\bullet}$ – C_6H_6, 60 %), 135 (100%);

HRMS (EI): calculated for $C_{20}H_{22}O_3$ $[M]^{+\bullet}$: 310.1569; found 310.1567.

(±)-Bicyclo[4.2.0]octadiene SI-4

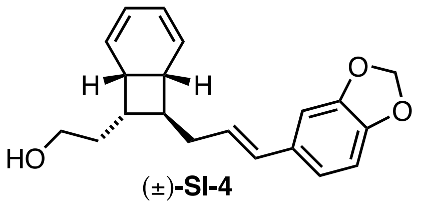

(±)-**SI-4**

Isolated as a pale yellow oil.

$\mathbf{R}_f$ = 0.19 (2:1 petroleum ether 40–60/Et_2O)

^{1}**H NMR** (400 MHz, $CDCl_3$) δ 6.88 (s, 1H), 6.73 (s, 2H), 6.30 (d, *J* = 15.6 Hz, 1H), 5.97 (dt, *J* = 14.8, 7.1 Hz, 1H), 5.93 (s, 2H), 5.86 (dd, *J* = 8.1, 7.0 Hz, 1H), 5.68 – 5.62 (m, 1H), 5.61 – 5.33 (m, 2H), 3.62 (t, *J* = 6.7 Hz, 2H), 3.17 (br t, *J* = 9.9 Hz, 1H), 2.64 – 2.52 (m, 2H), 2.51 – 2.39 (m, 1H), 2.39 – 2.20 (m, 2H), 1.96 – 1.75 (m, 2H) ppm;

^{13}**C NMR** (100 MHz, $CDCl_3$) δ 148.1 (C_q), 146.8 (C_q), 132.4 (C_q), 130.5 (CH), 127.3 (CH), 127.2 (CH), 126.4 (CH), 124.4 (CH), 121.6 (CH), 120.5 (CH), 108.4 (CH), 105.5 (CH), 101.1 (CH_2), 61.9 (CH_2), 51.8 (CH), 47.1 (CH), 39.4 (CH_2), 36.8 (CH), 34.2 (CH), 33.8 (CH_2) ppm;

IR (thin film): ν_{max} = 3375, 2927, 1793, 1487, 1096 cm^{-1};

MS (EI): *m/z* (%): 310 ($M^{+\bullet}$, 15 %), 232 ($M^{+\bullet}$ – C_6H_6, 60 %), 135 (100%);

HRMS (EI): calculated for $C_{20}H_{22}O_3$ $[M]^{+\bullet}$: 310.1569; found 310.1567.

Radical cation formal IMDA reaction of (±)-bicyclo[4.2.0]octadiene ***26***

1. Radical cation formal IMDA reaction using Ledwith–Weitz salt 27

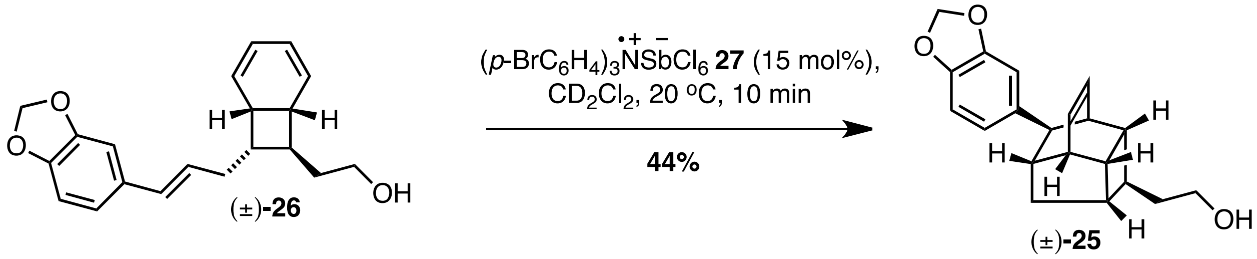

An NMR tube was charged with (±)-bicyclo[4.2.0]octadiene **26** (9.05 μmol by ^{1}H NMR analysis using 1,2-dichloroethane as an internal standard, 1.0 mol. equiv.) and CD_2Cl_2 (0.50 ml). Tris(4-bromophenyl)ammoniumyl hexachloroantimonate **27** (1.1 mg, 1.34 μmol, 15 mol%) was then added, the NMR tube capped and shaken at ambient room temperature (20 °C) for 10 min. After this time ^{1}H NMR analysis found the IMDA reaction to be complete with alcohol **(±)-25** observed as the major component (4.00 μmol by ^{1}H NMR analysis using 1,2-dichloroethane as an internal standard, 44%).

2. Visible light photoredox catalysed radical cation formal IMDA reaction

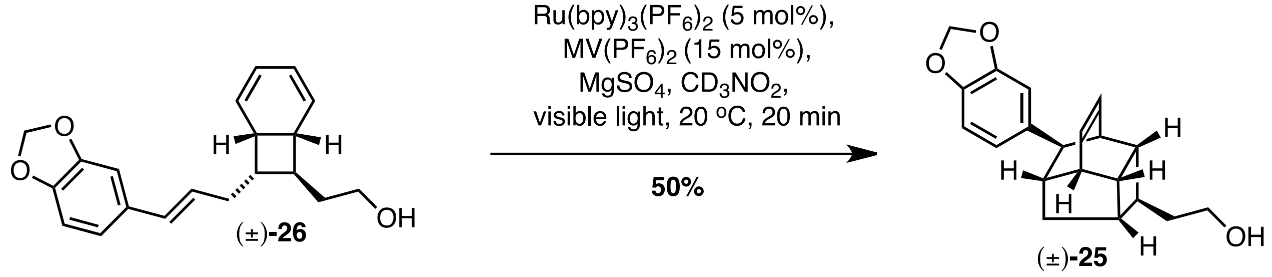

This protocol was based on the visible light catalysed radical cation cycloaddition conditions developed by Yoon and co-workers.[10] $Ru(bpy)_3(PF_6)_2$ and $MV(PF_6)_2$ were prepared as described by Yoon *et al.*[10]

An NMR tube was charged with (±)-bicyclo[4.2.0]octadiene **26** (7.90 μmol by ^{1}H NMR analysis using 1,2-dichloroethane as an internal standard, 1.0 mol. equiv.) and CD_3NO_2 (0.50 ml). Anhydrous $MgSO_4$ (5.0 mg, 200 w/w%), $Ru(bpy)_3(PF_6)_2$ (0.40 mg, 0.46 μmol, 5 mol%) and $MV(PF_6)_2$ (0.60 mg, 1.3 μmol, 15 mol%) were then added, the NMR tube capped and shaken at ambient room temperature (20 °C) in front of a 23 W compact fluorescent light bulb for 20 min. After this time ^{1}H NMR analysis found the IMDA reaction to be complete with alcohol **(±)-25** observed as the major component (3.95 μmol by ^{1}H NMR analysis using 1,2-dichloroethane as an internal standard, 50%).

References

[1] A. B. Pangborn, M. A. Giardello, R. H. Grubbs, R. K. Rosen and F. J. Timmers, *Organometallics,* 1996, **15**, 1518.

[2] D. D. Perrin and W. L. F. Armarego, *Purification of Laboratory Chemicals*, Pergamon, Oxford, 1988.

[3] H. F. Sneddon and M. J. Gaunt, S. V. Ley, *Org. Lett.,* 2003, **5**, 1147.

[4] D. D. Kim, S. J. Lee and P. Beak, *J. Org. Chem.,* 2005, **70**, 5376.

[5] P. S.-W. Leung, Y. Teng and P. H. Toy, *Org. Lett.,* 2010, **12**, 4996.

[6] D. Imperio, T. Pirali, U. Galli, F. Pagliai, L. Cafici, P. L. Canonico, G. Sorba, A. A. Genazzani and G. C. Tron, *Bioorg. Med. Chem.,* 2007, **15**, 6748.

[7] C. M. Williams and L. N. Mander, *Tetrahedron,* 2001, **57**, 425.

[8] M. Azmi, C. Gény, A. Leverrier, M. Litaudon, V. Dumontet, N. Birlirakis, F. Guéritte, K. H. Leong, S. N. A. Halim, K. Mohamad and K. Awang, *Molecules,* 2014, **19**, 1732.

[9] S. L. Drew, A. L. Lawrence, M. S. Sherburn, *Angew. Chem.* 2013, **125**, 4315; *Angew. Chem. Int. Ed.* 2013, **52**, 4221.

[10] S. Lin, C. E. Padilla, M. A. Ischay and T. P. Yoon*, Tetrahedron Lett*. 2012, **53**, 3073.

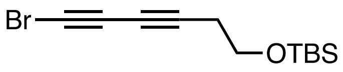

11

400 MHz ^{1}H NMR ($CDCl_3$)

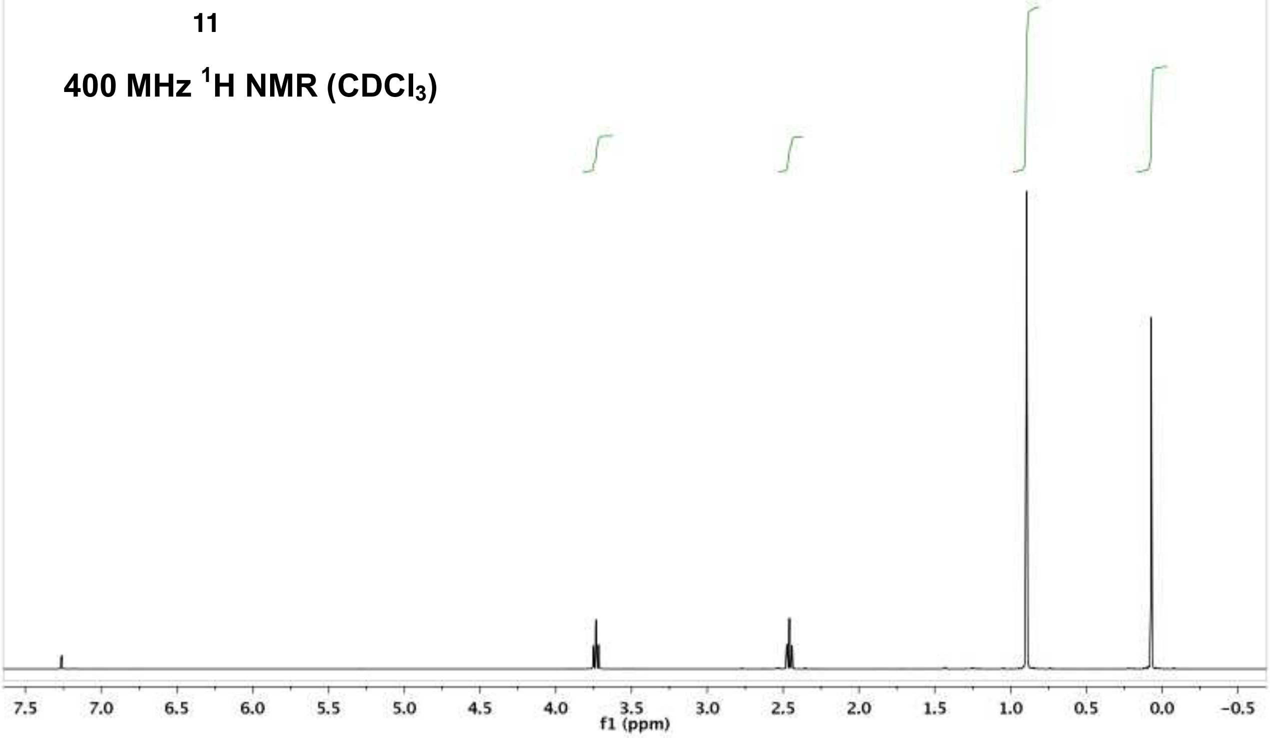

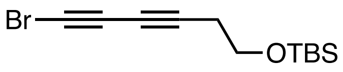

11

100 MHz ^{13}C NMR ($CDCl_3$)

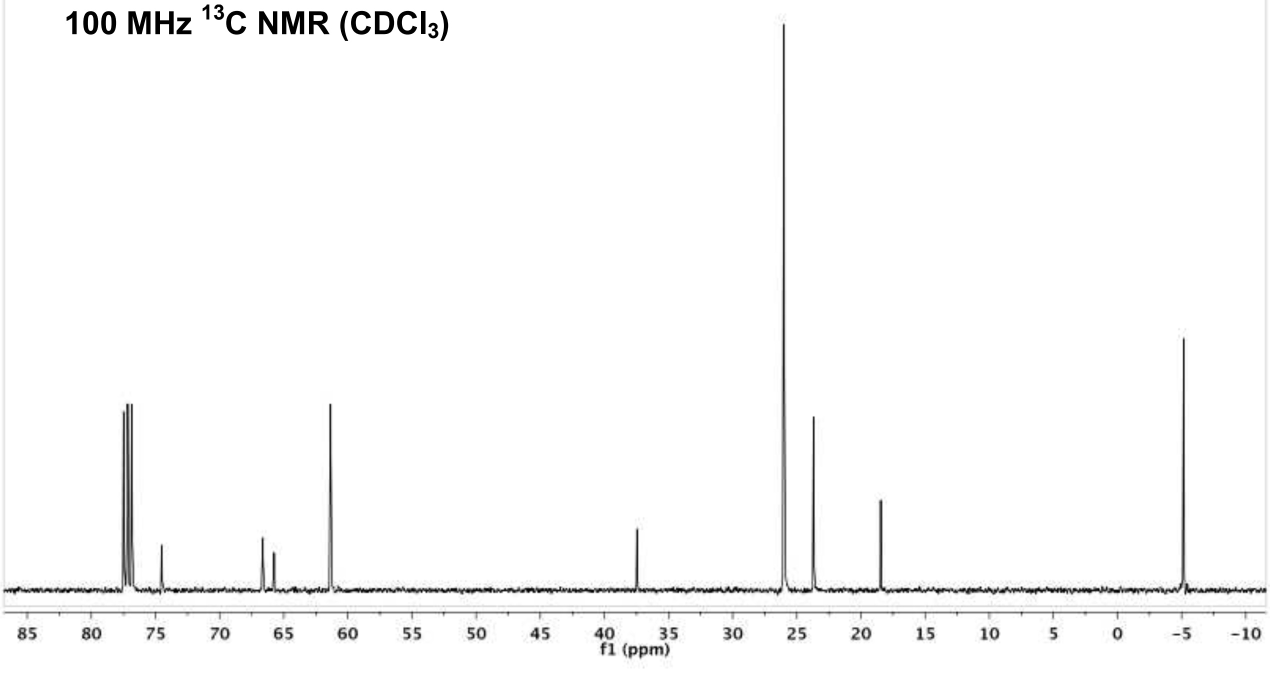

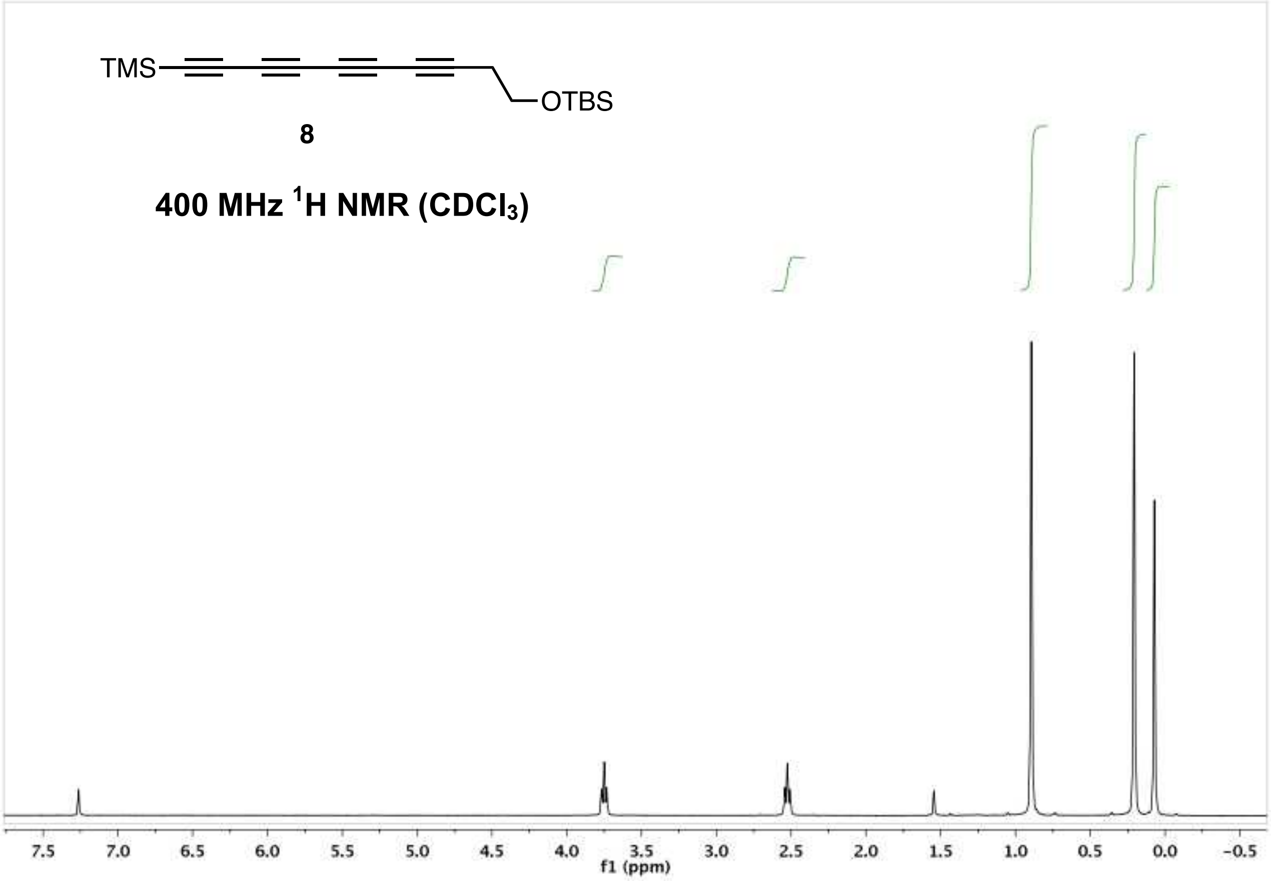
TMS
OTBS
8
400 MHz 1H NMR (CDCl3)
f1 (ppm)

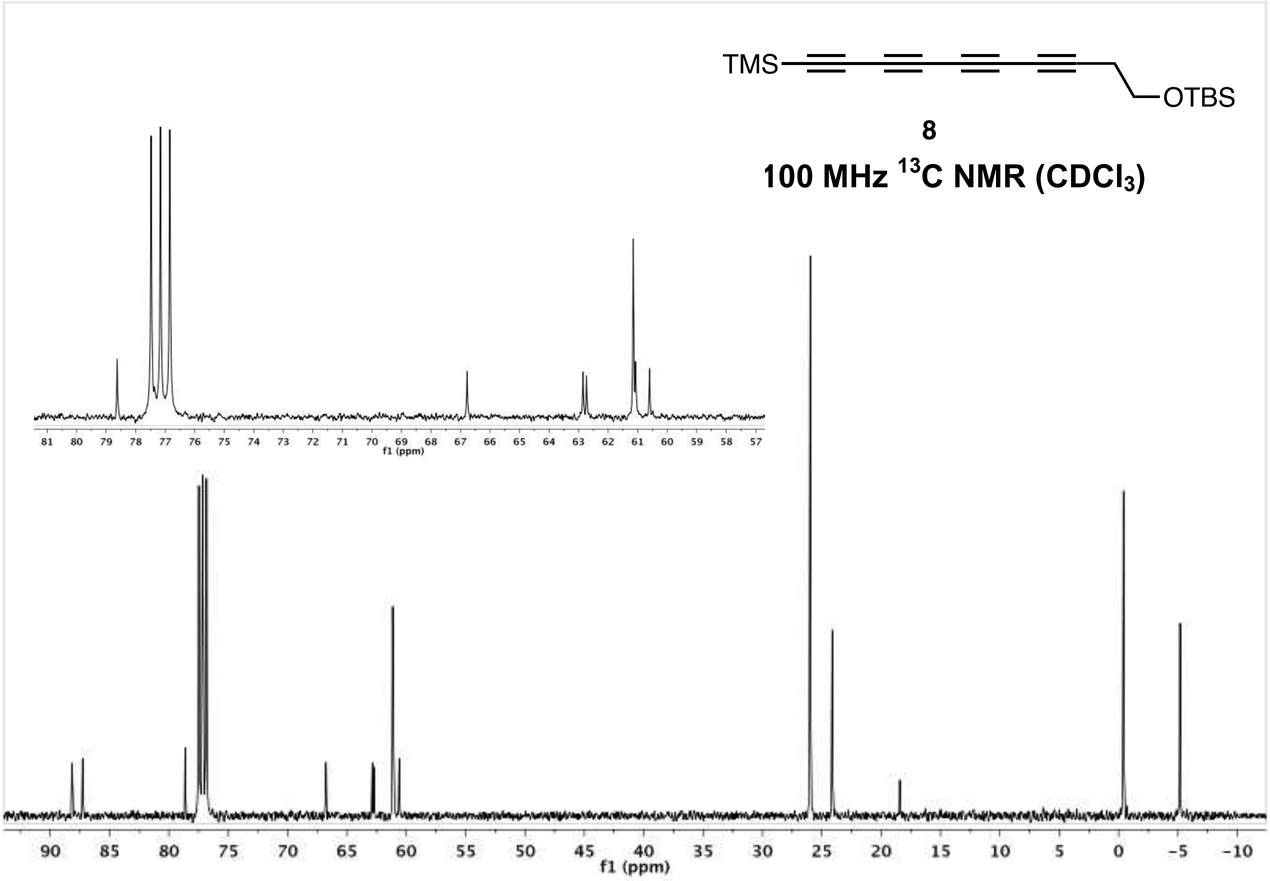
TMS
OTBS
8
100 MHz 13C NMR (CDCl3)
f1 (ppm)

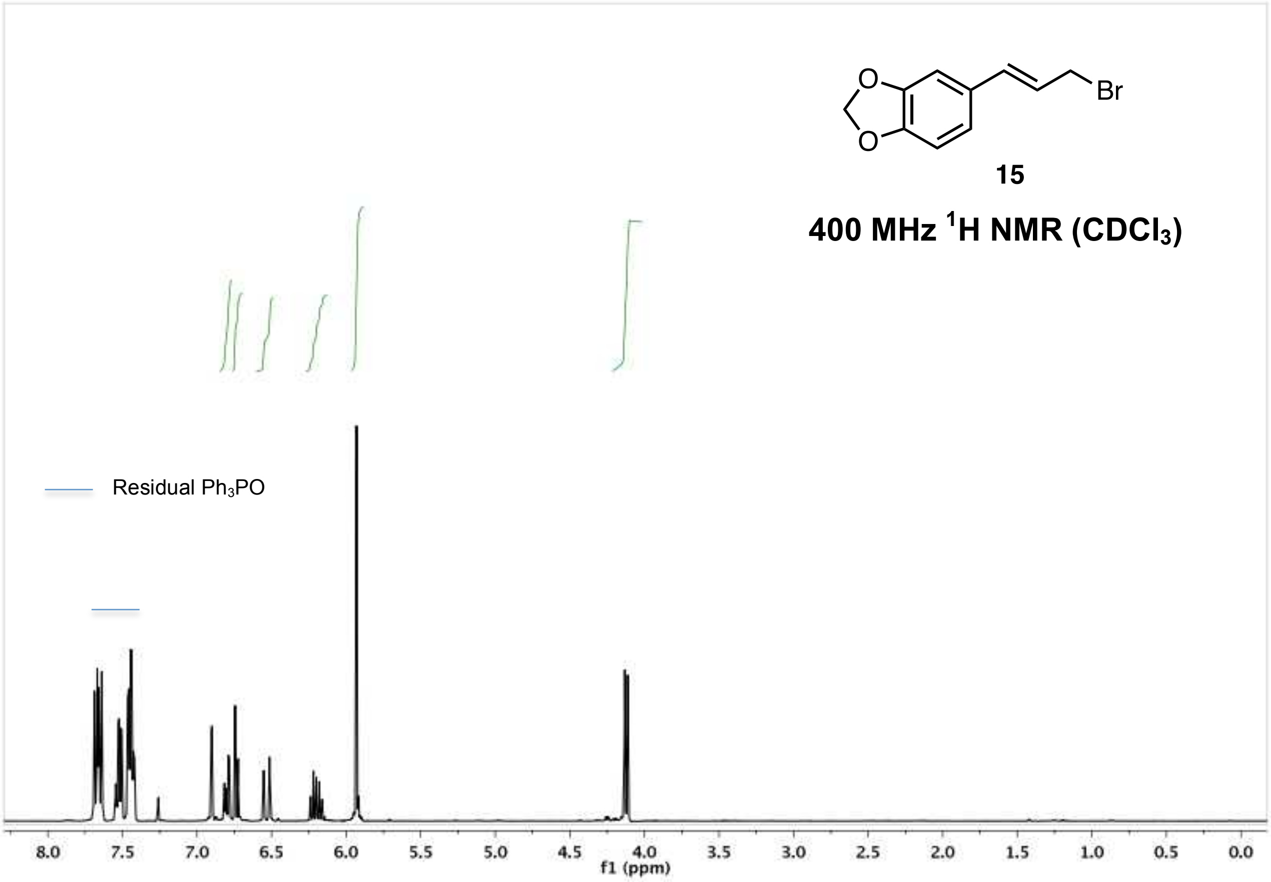
Br
15
400 MHz 1H NMR (CDCl3)
Residual Ph3PO
f1 (ppm)

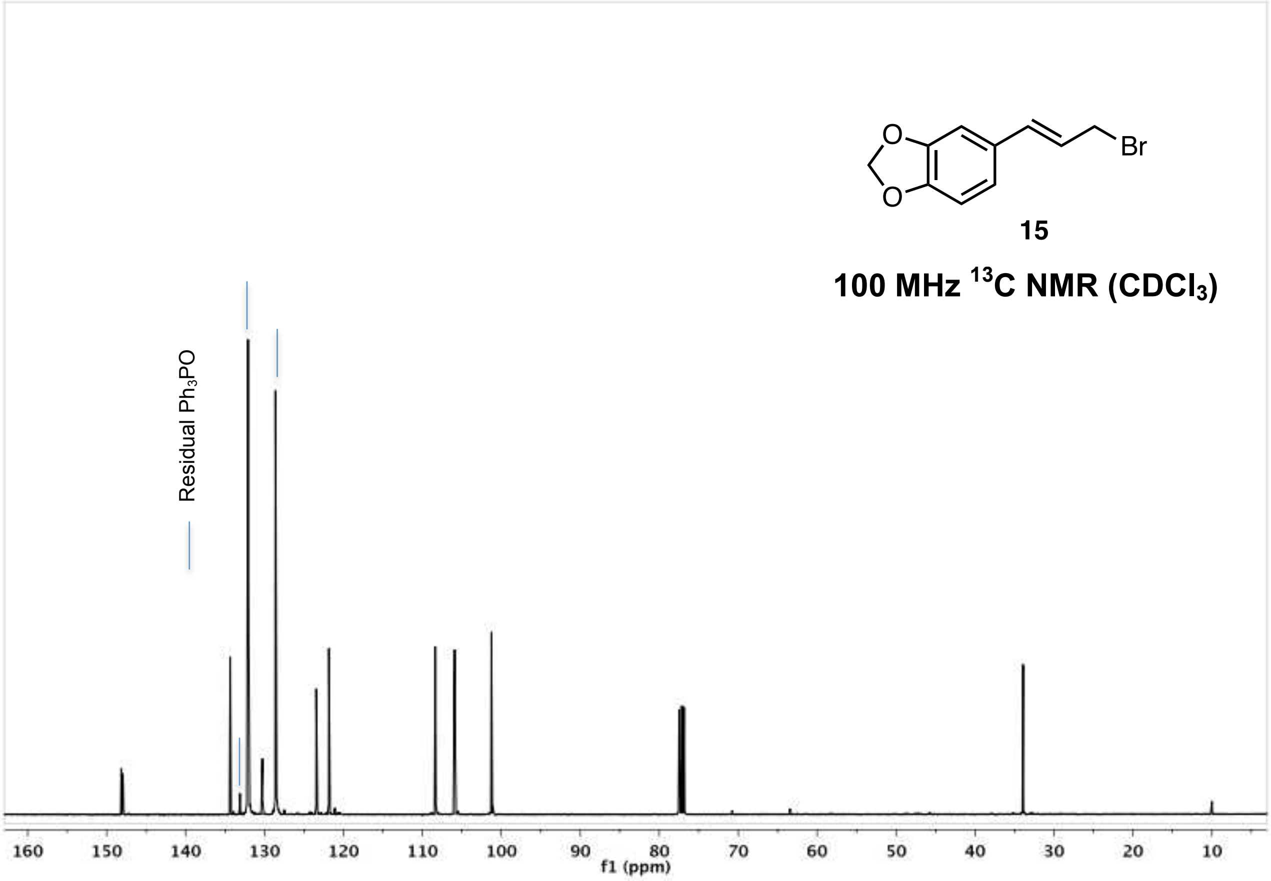
Br
15
100 MHz 13C NMR (CDCl3)
Residual Ph3PO
f1 (ppm)

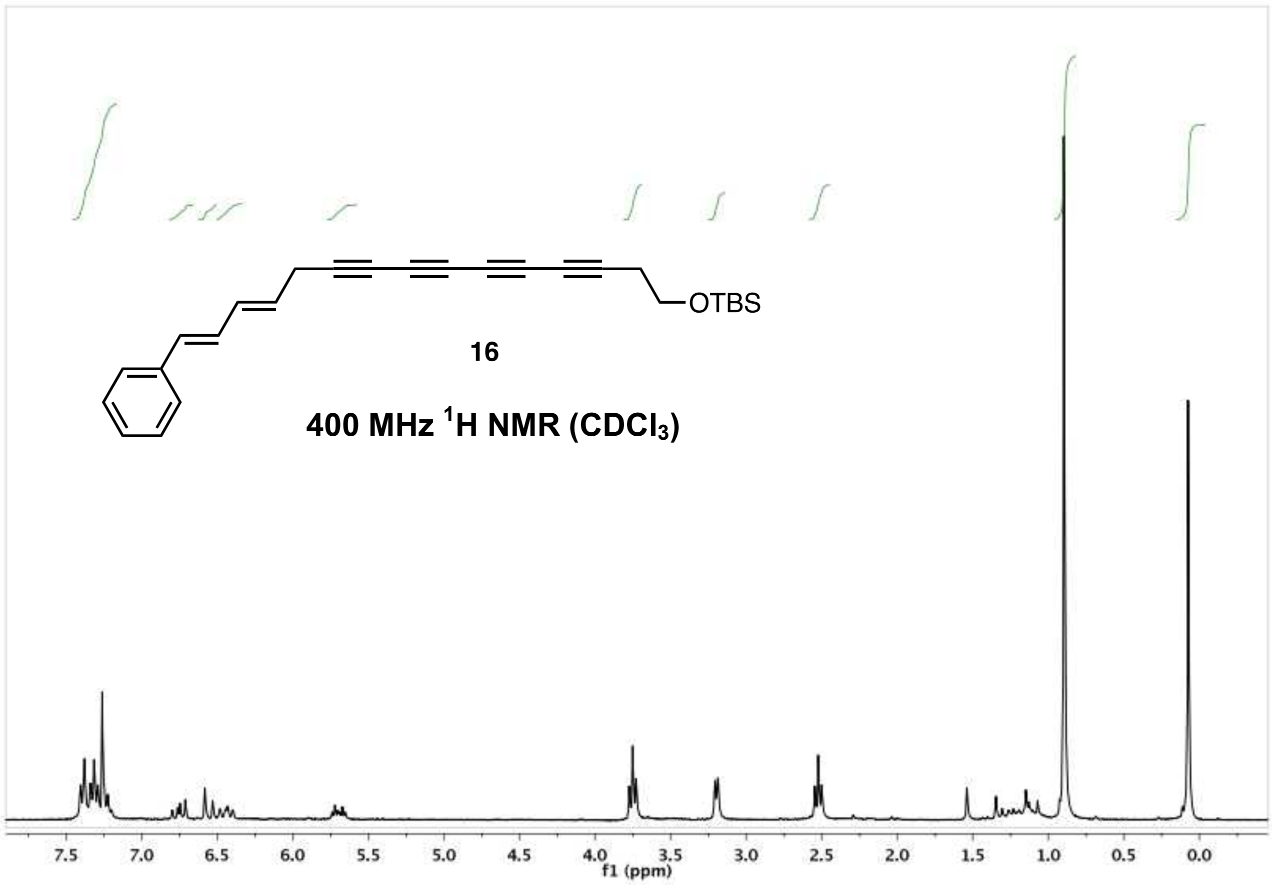
OTBS
16
400 MHz ¹H NMR (CDCl₃)
f1 (ppm)

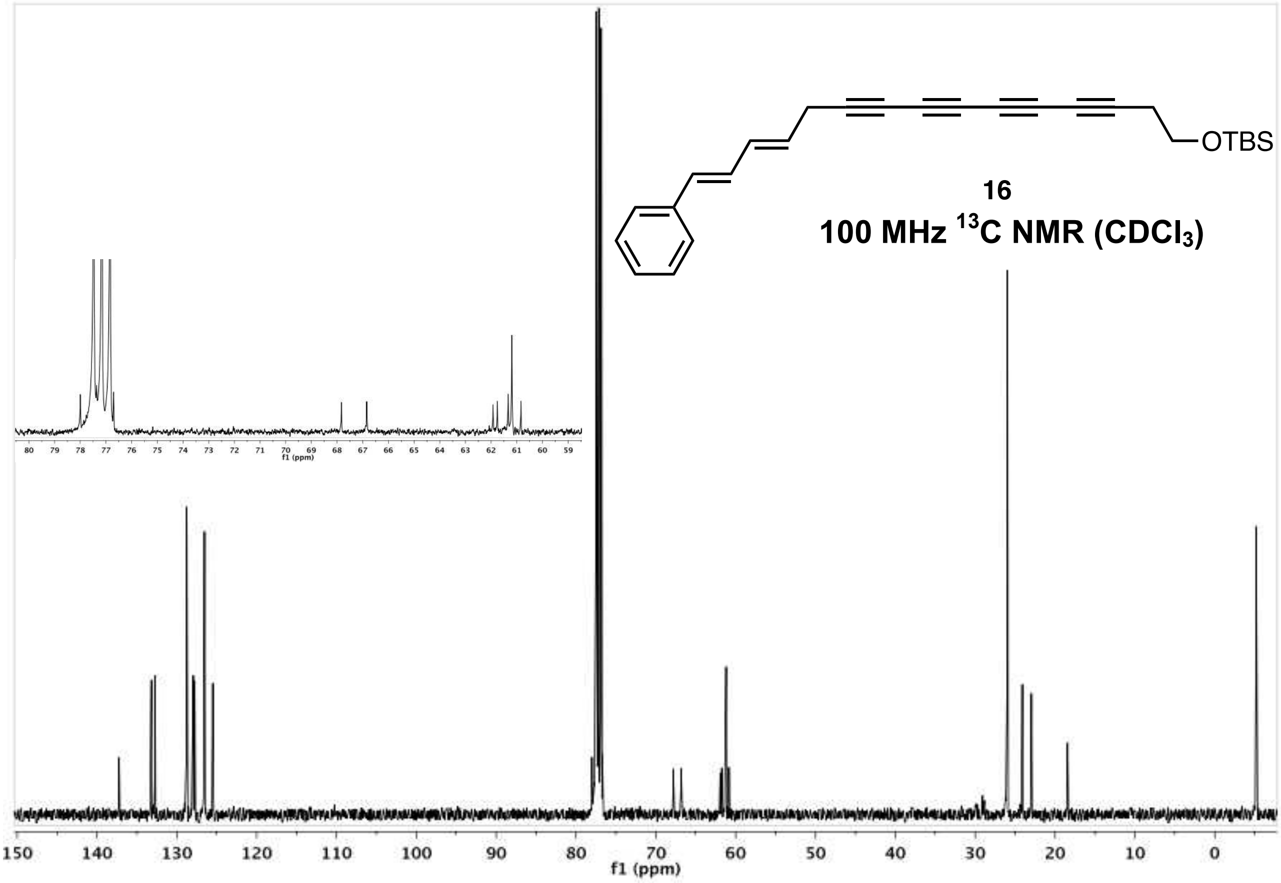
OTBS
16
100 MHz ¹³C NMR (CDCl₃)
f1 (ppm)

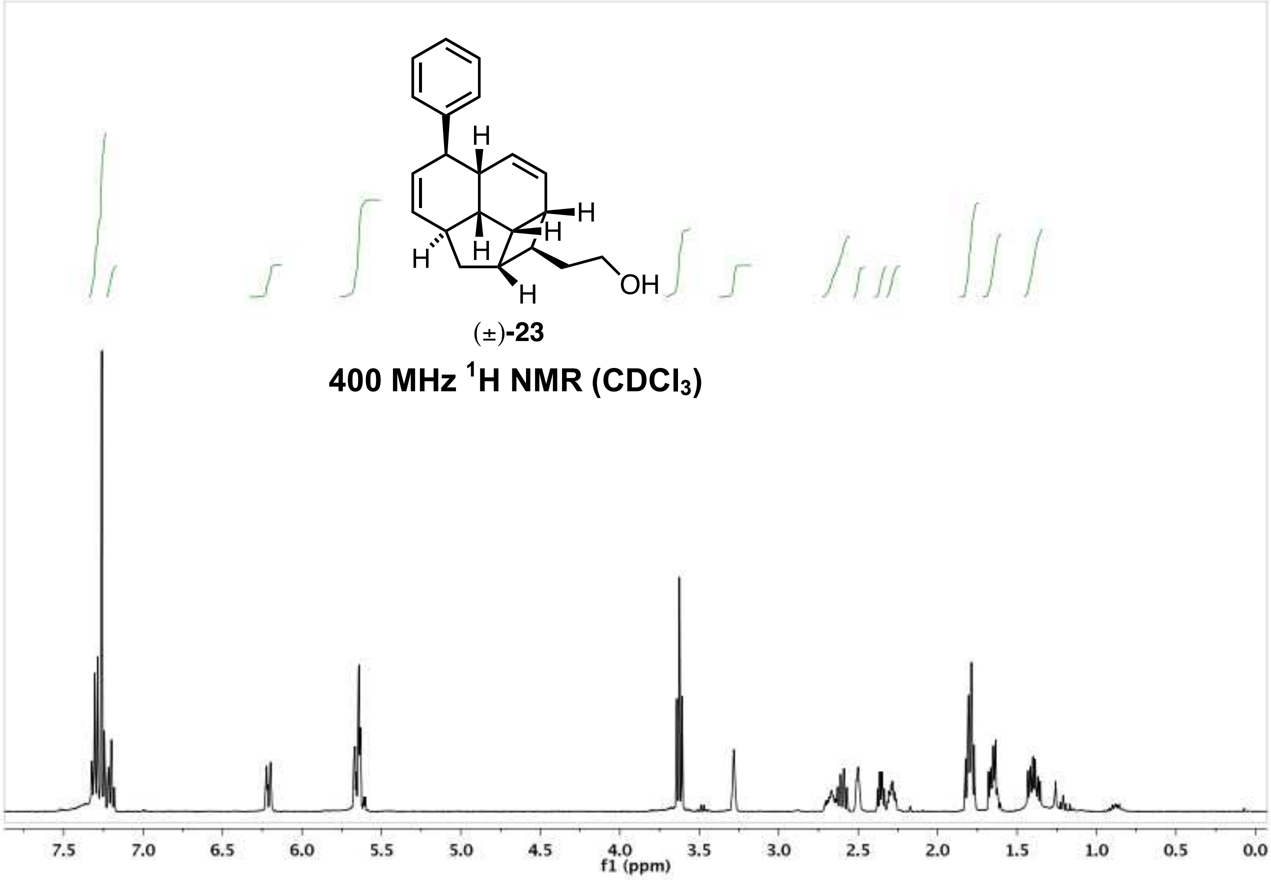
H
H
H
H
H
H
OH
(±)-23
400 MHz 1H NMR (CDCl3)
7.5 7.0 6.5 6.0 5.5 5.0 4.5 4.0 3.5 3.0 2.5 2.0 1.5 1.0 0.5 0.0
f1 (ppm)

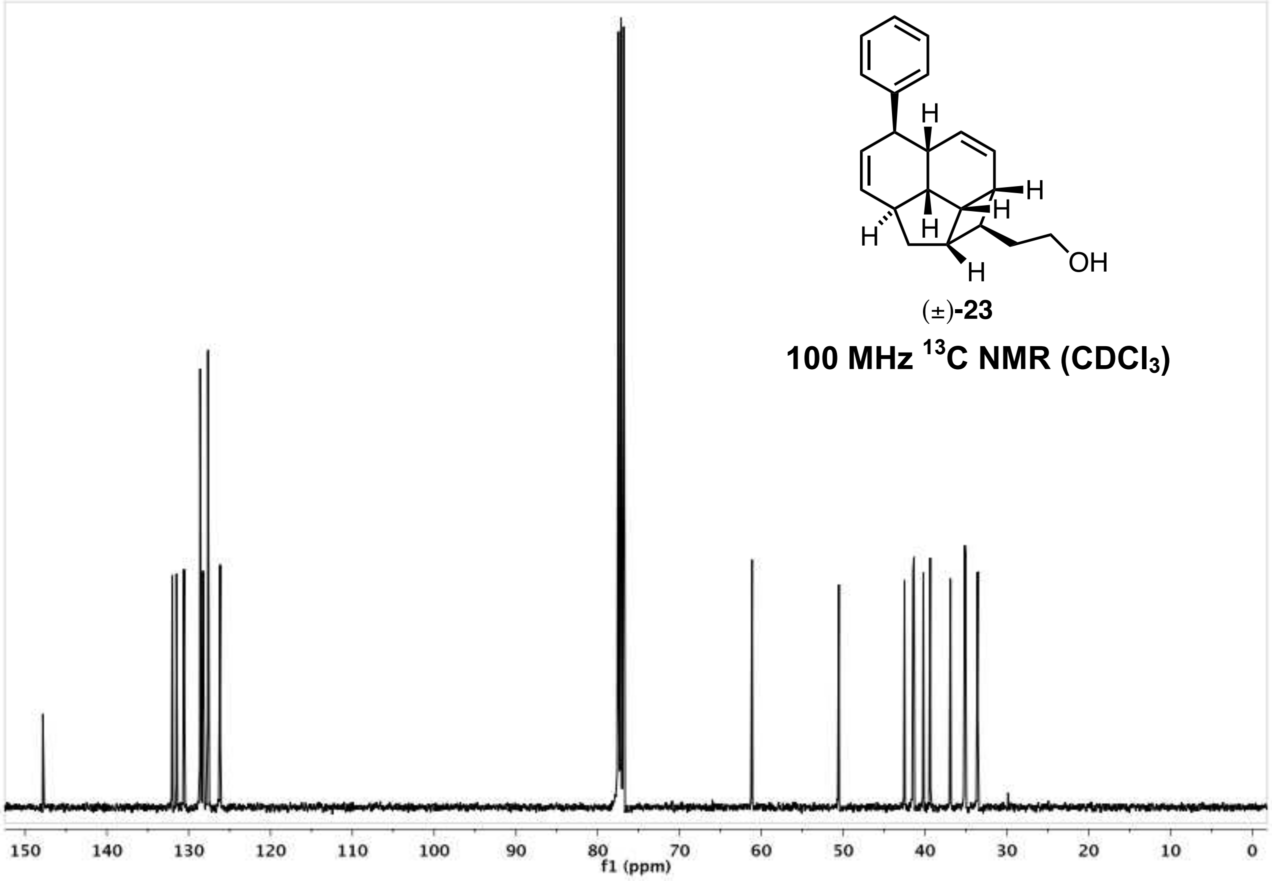
H
H
H
H
H
H
OH
(±)-23
100 MHz 13C NMR (CDCl3)
150 140 130 120 110 100 90 80 70 60 50 40 30 20 10 0
f1 (ppm)

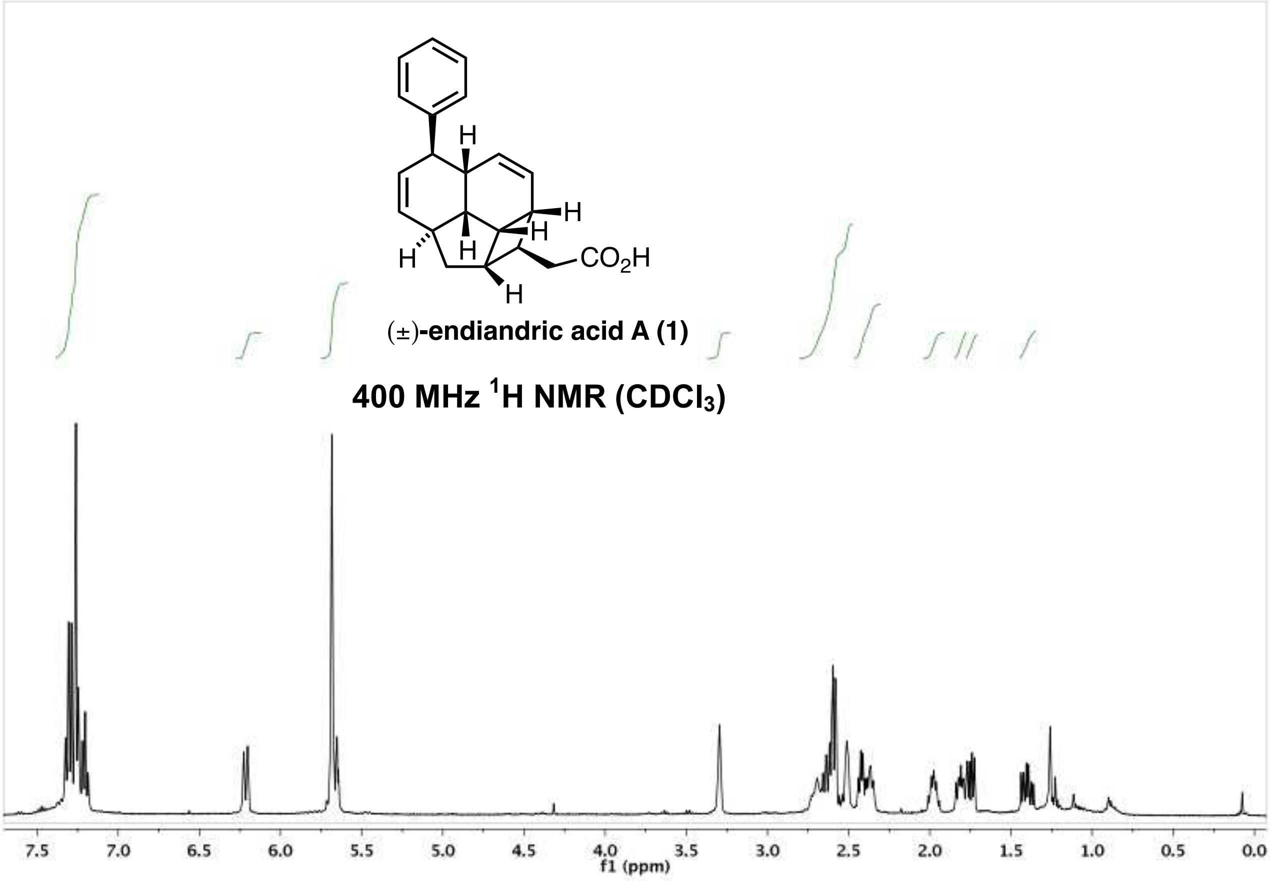
H
H
H
H
H
CO_2H
H
(±)-endiandric acid A (1)
400 MHz ^{1}H NMR ($CDCl_3$)
f1 (ppm)

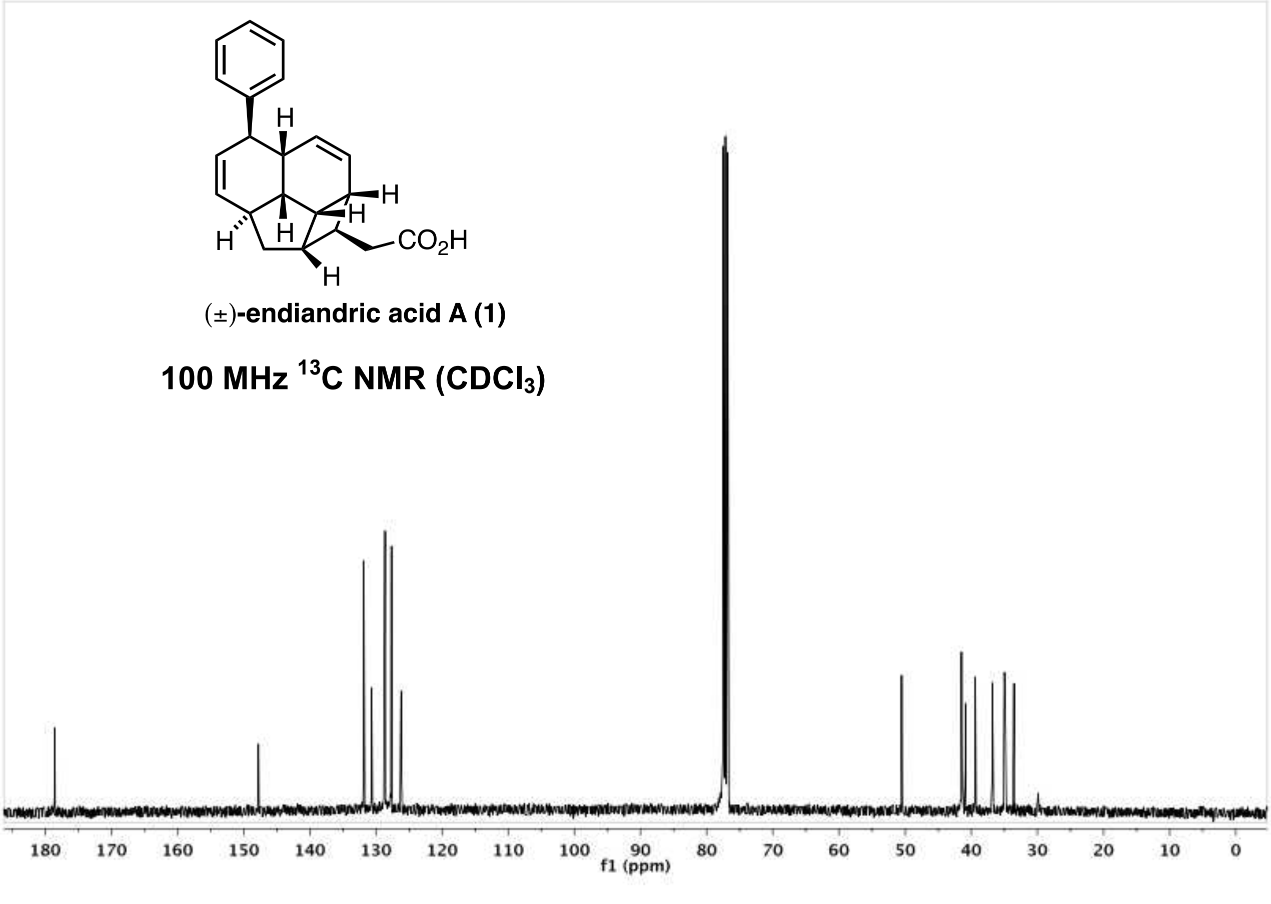
H
H
H
H
H
CO_2H
H
(±)-endiandric acid A (1)
100 MHz ^{13}C NMR ($CDCl_3$)
f1 (ppm)

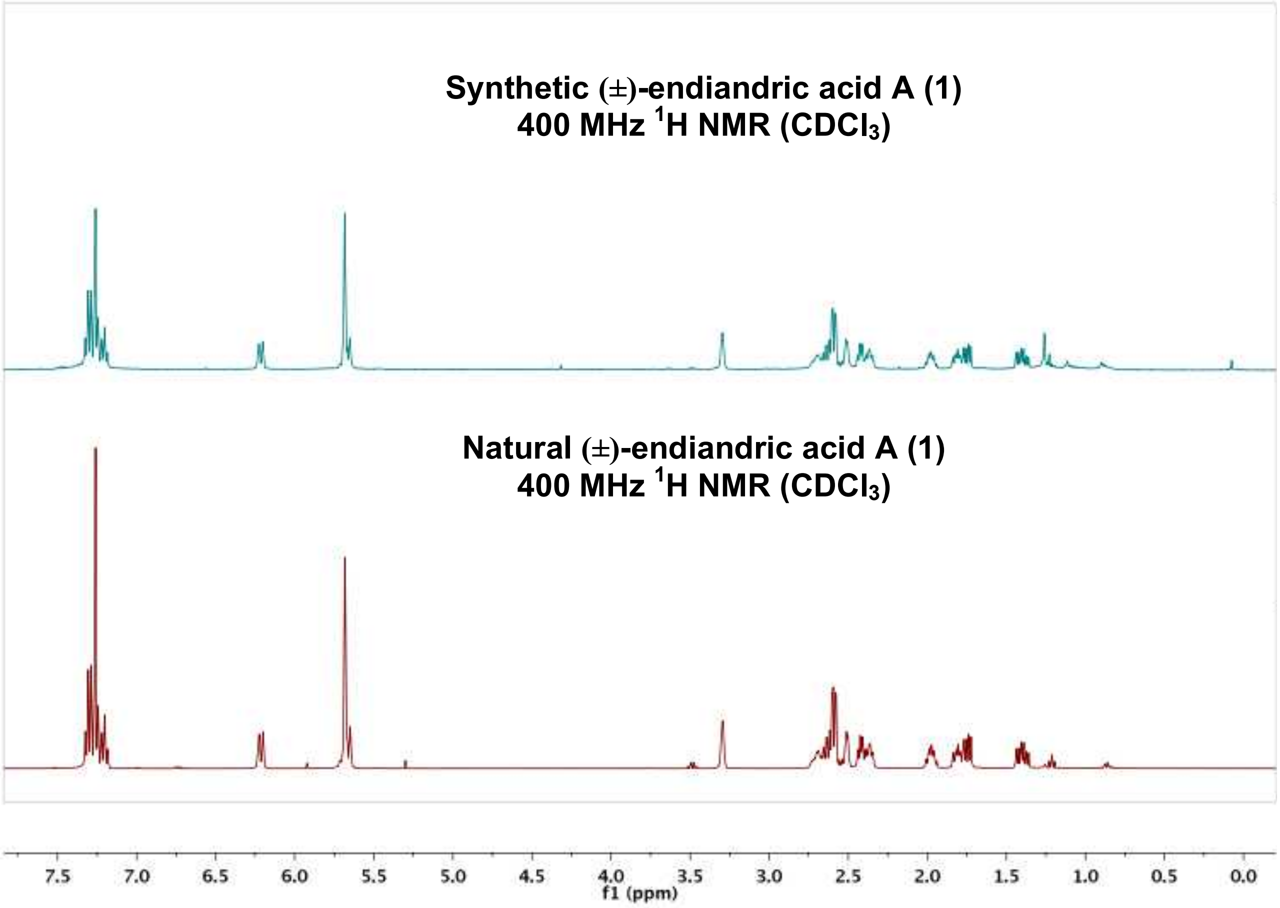
Synthetic (±)-endiandric acid A (1)
400 MHz 1H NMR (CDCl3)
Natural (±)-endiandric acid A (1)
400 MHz 1H NMR (CDCl3)
7.5 7.0 6.5 6.0 5.5 5.0 4.5 4.0 3.5 3.0 2.5 2.0 1.5 1.0 0.5 0.0
f1 (ppm)

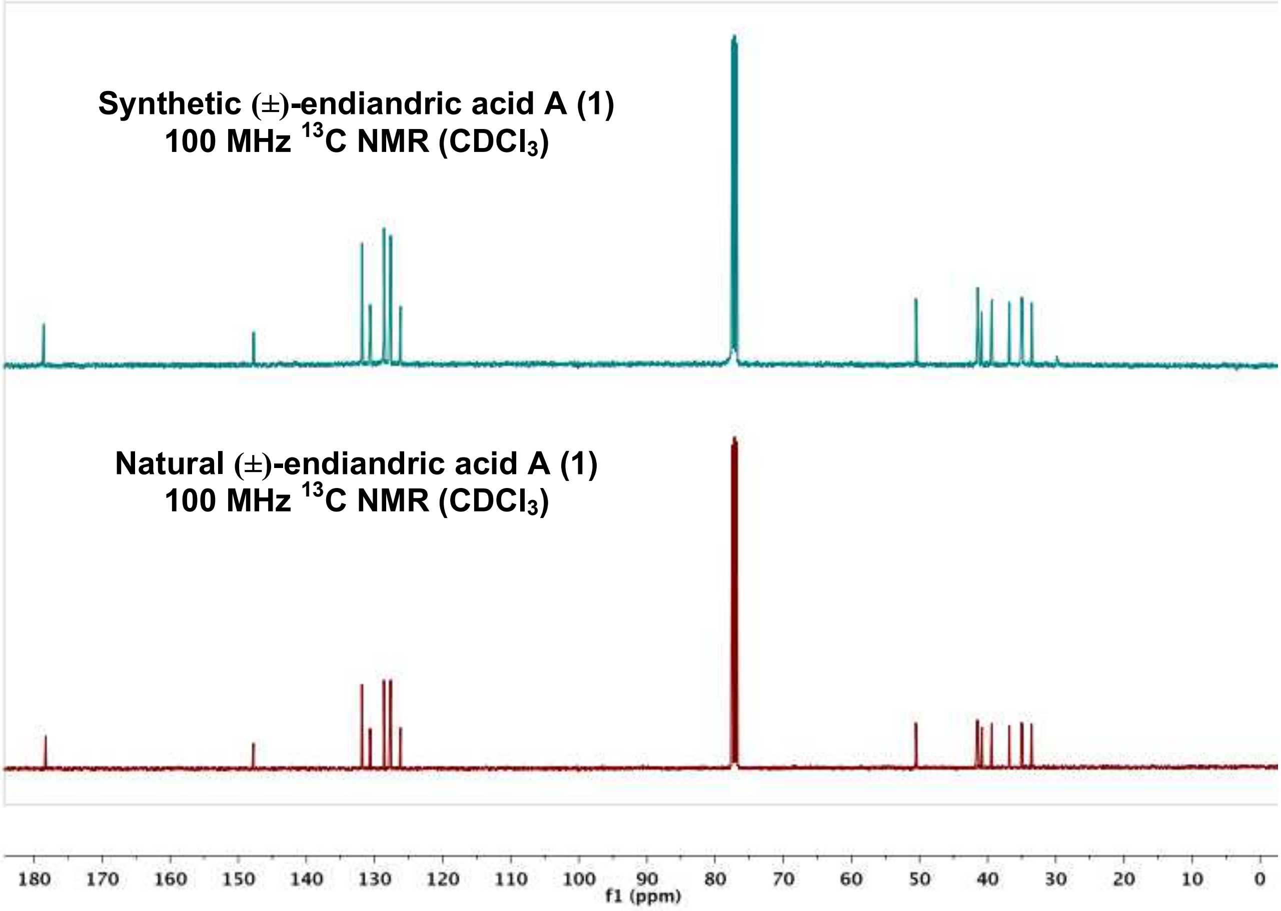
Synthetic (±)-endiandric acid A (1)
100 MHz 13C NMR (CDCl3)
Natural (±)-endiandric acid A (1)
100 MHz 13C NMR (CDCl3)
180 170 160 150 140 130 120 110 100 90 80 70 60 50 40 30 20 10 0
f1 (ppm)

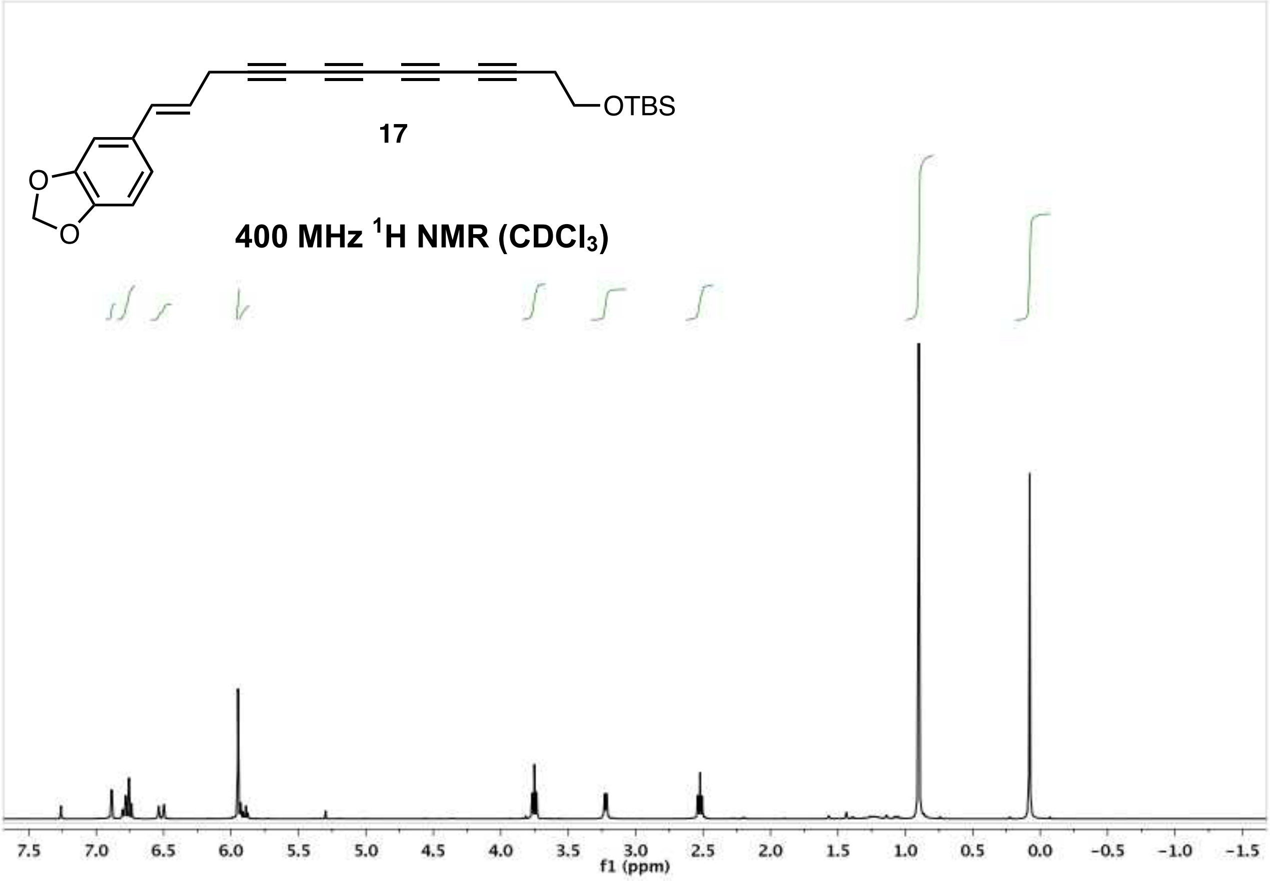

OTBS
17
O
O
400 MHz ^{1}H NMR ($CDCl_3$)
f1 (ppm)

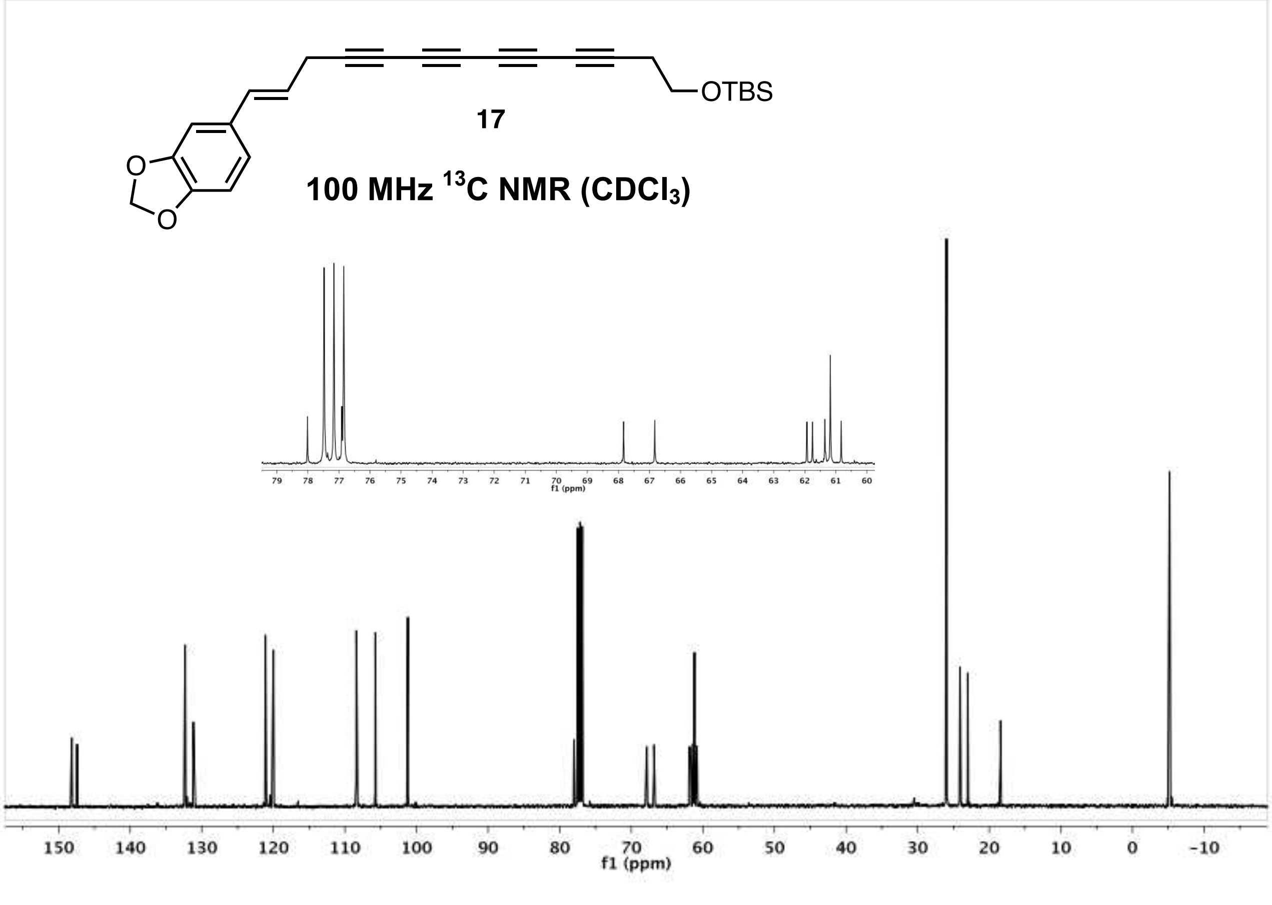

OTBS
17
O
O
100 MHz ^{13}C NMR ($CDCl_3$)
f1 (ppm)
f1 (ppm)

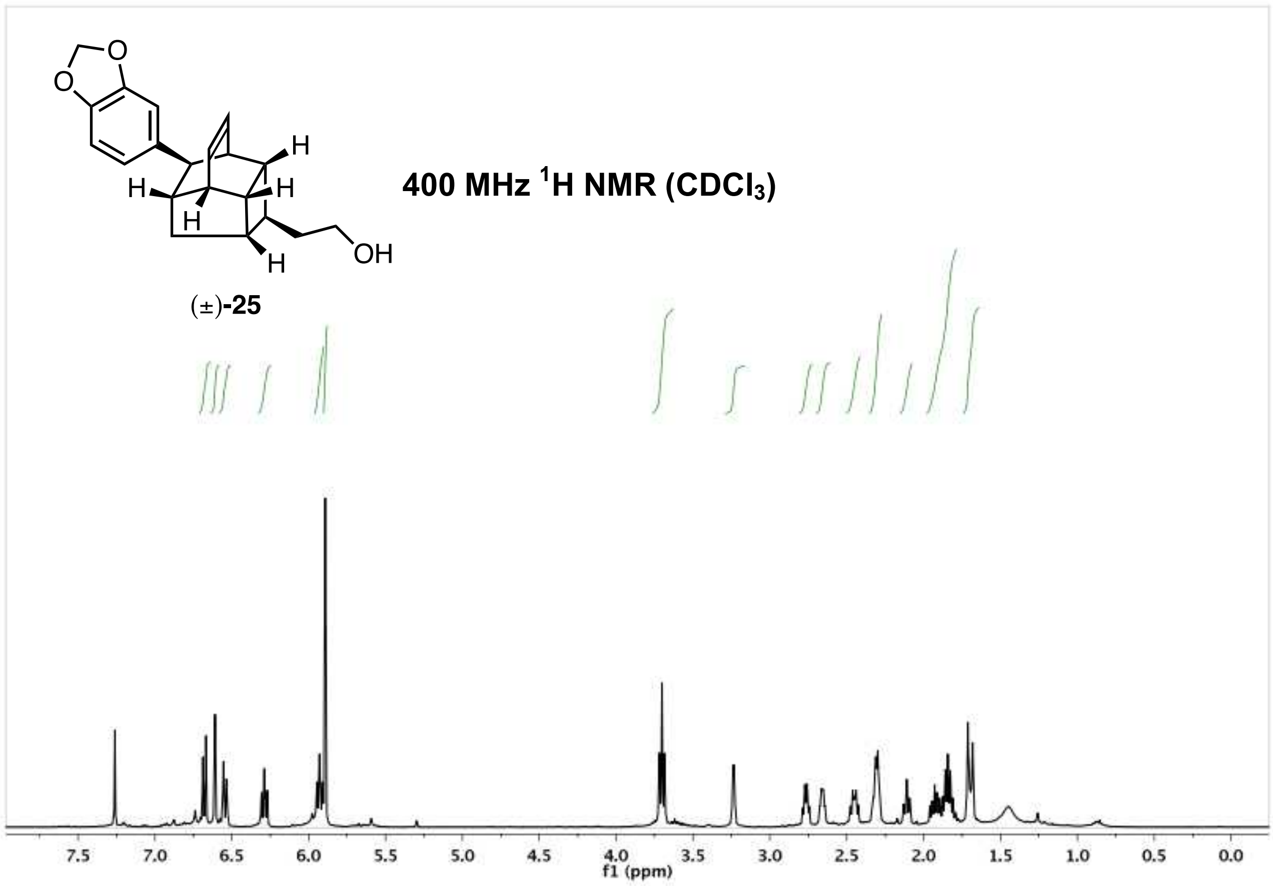

H
H
H
H
H
OH
O
O
(±)-25
400 MHz ^{1}H NMR ($CDCl_3$)
7.5 7.0 6.5 6.0 5.5 5.0 4.5 4.0 3.5 3.0 2.5 2.0 1.5 1.0 0.5 0.0
f1 (ppm)

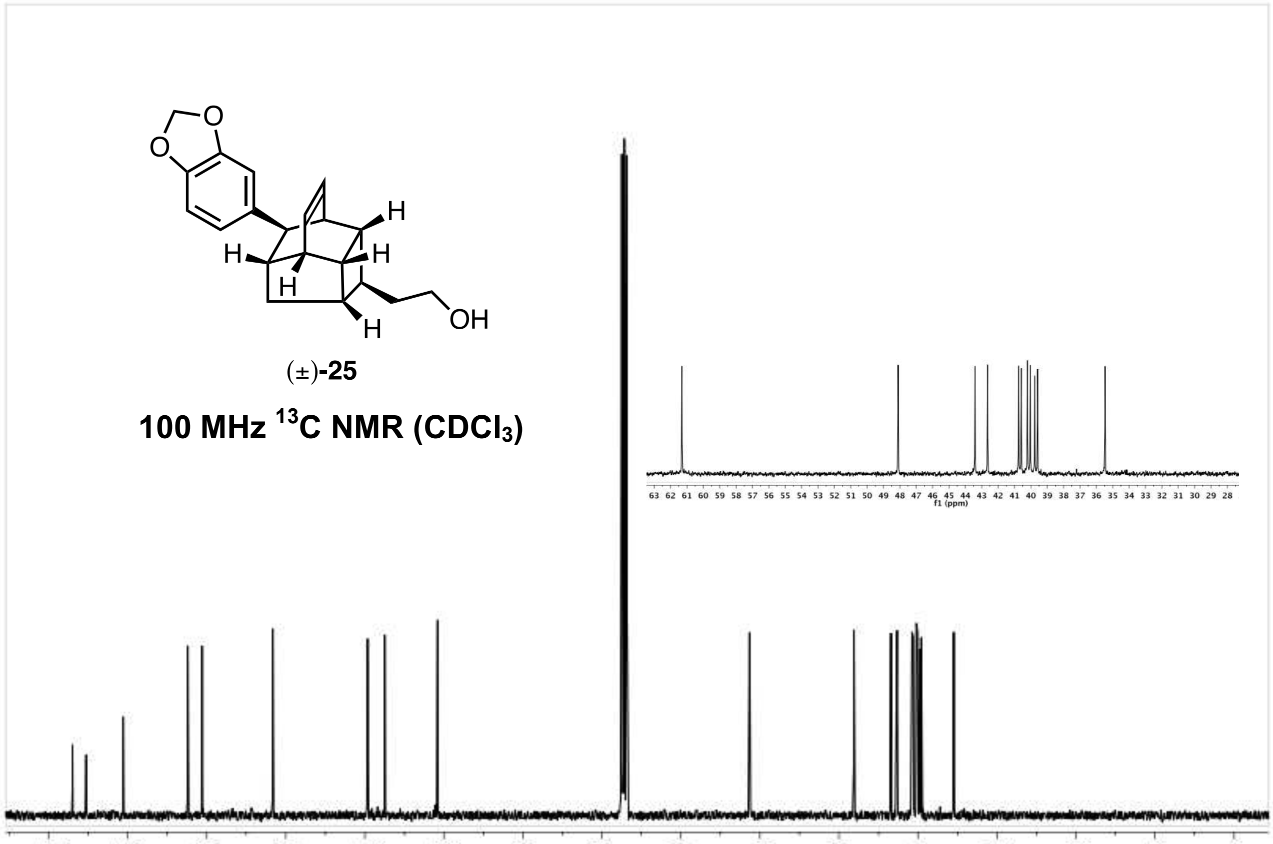

H
H
H
H
H
OH
O
O
(±)-25
100 MHz ^{13}C NMR ($CDCl_3$)
150 140 130 120 110 100 90 80 70 60 50 40 30 20 10 0
f1 (ppm)

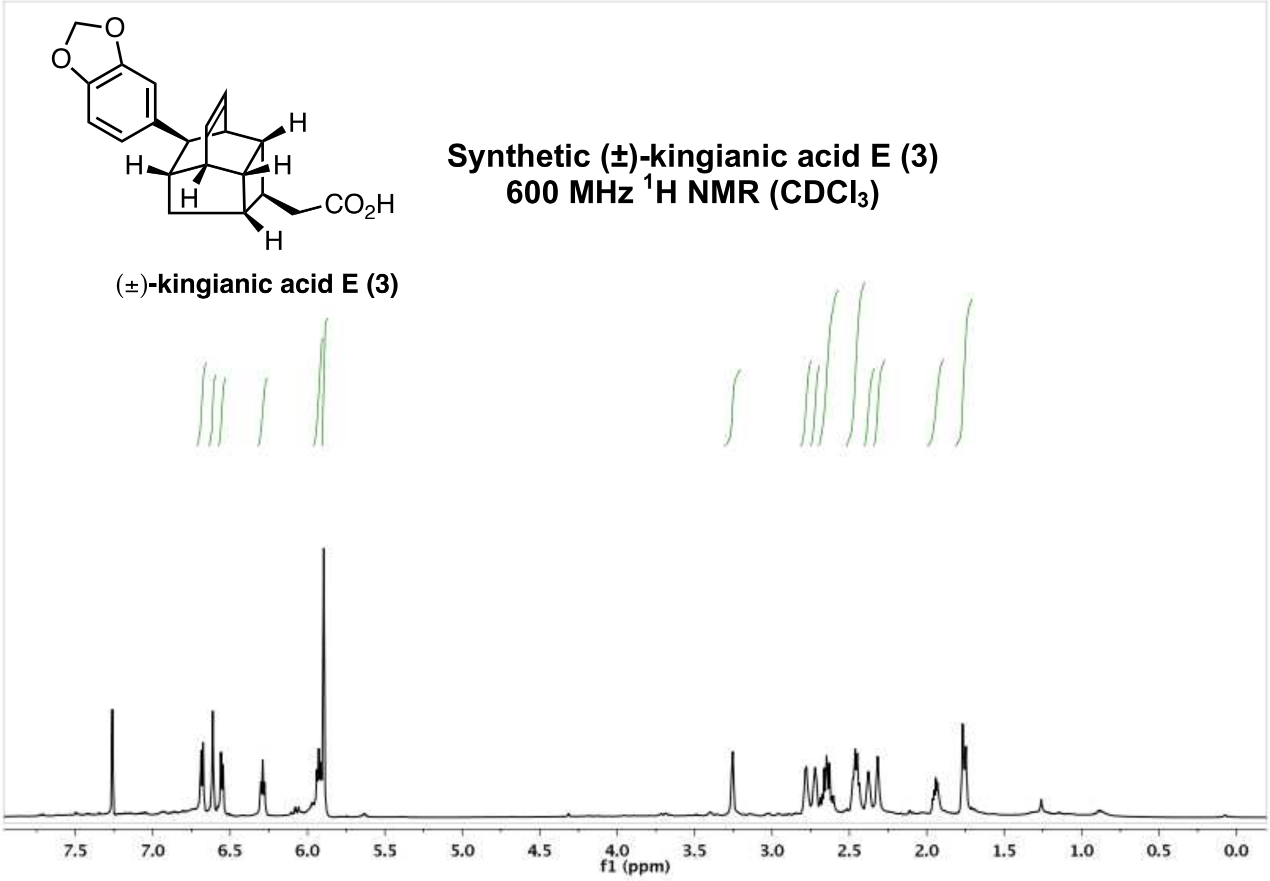

(±)-**kingianic acid E (3)**

Synthetic (±)-kingianic acid E (3)
600 MHz ^{1}H NMR ($CDCl_3$)

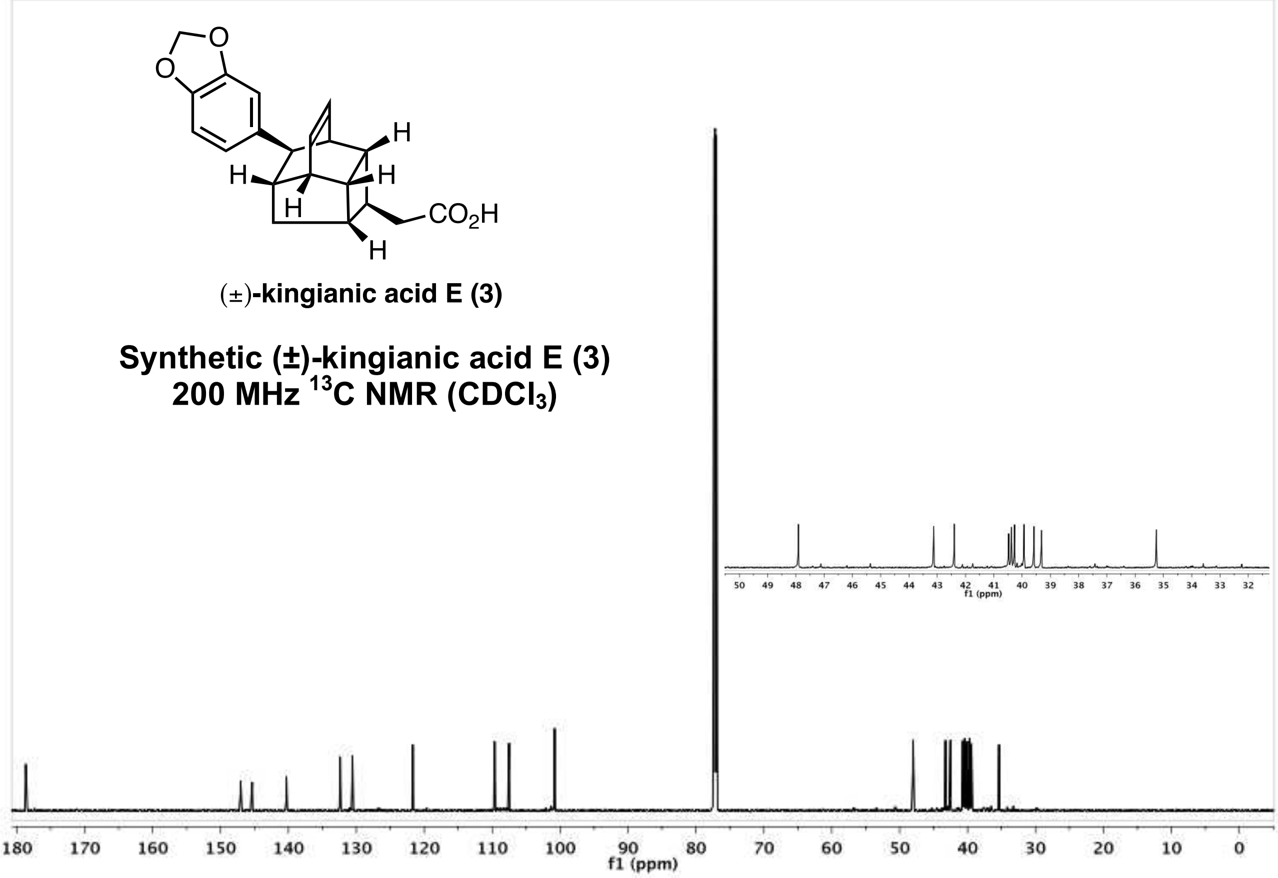

(±)-**kingianic acid E (3)**

Synthetic (±)-kingianic acid E (3)
200 MHz ^{13}C NMR ($CDCl_3$)

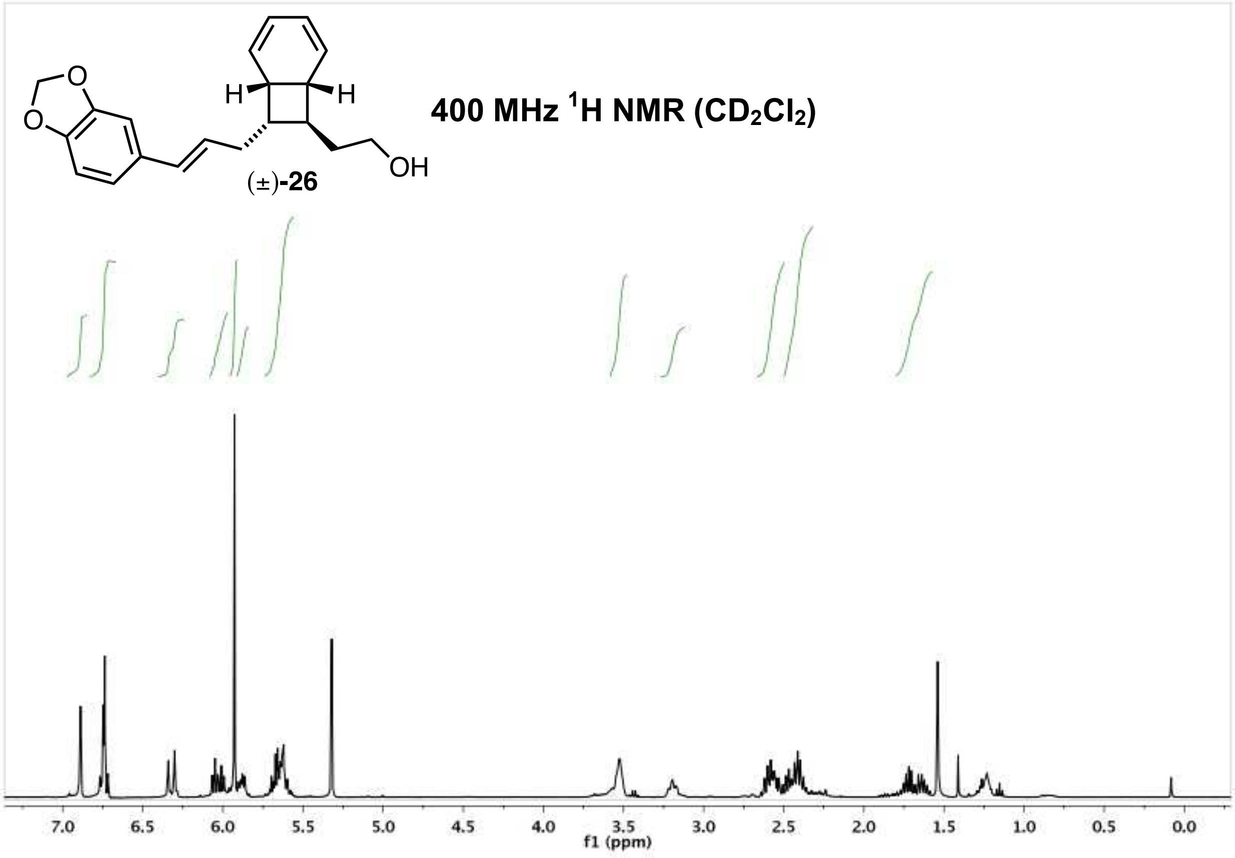
H
H
OH
O
O
(±)-26
400 MHz 1H NMR (CD2Cl2)
7.0 6.5 6.0 5.5 5.0 4.5 4.0 3.5 3.0 2.5 2.0 1.5 1.0 0.5 0.0
f1 (ppm)

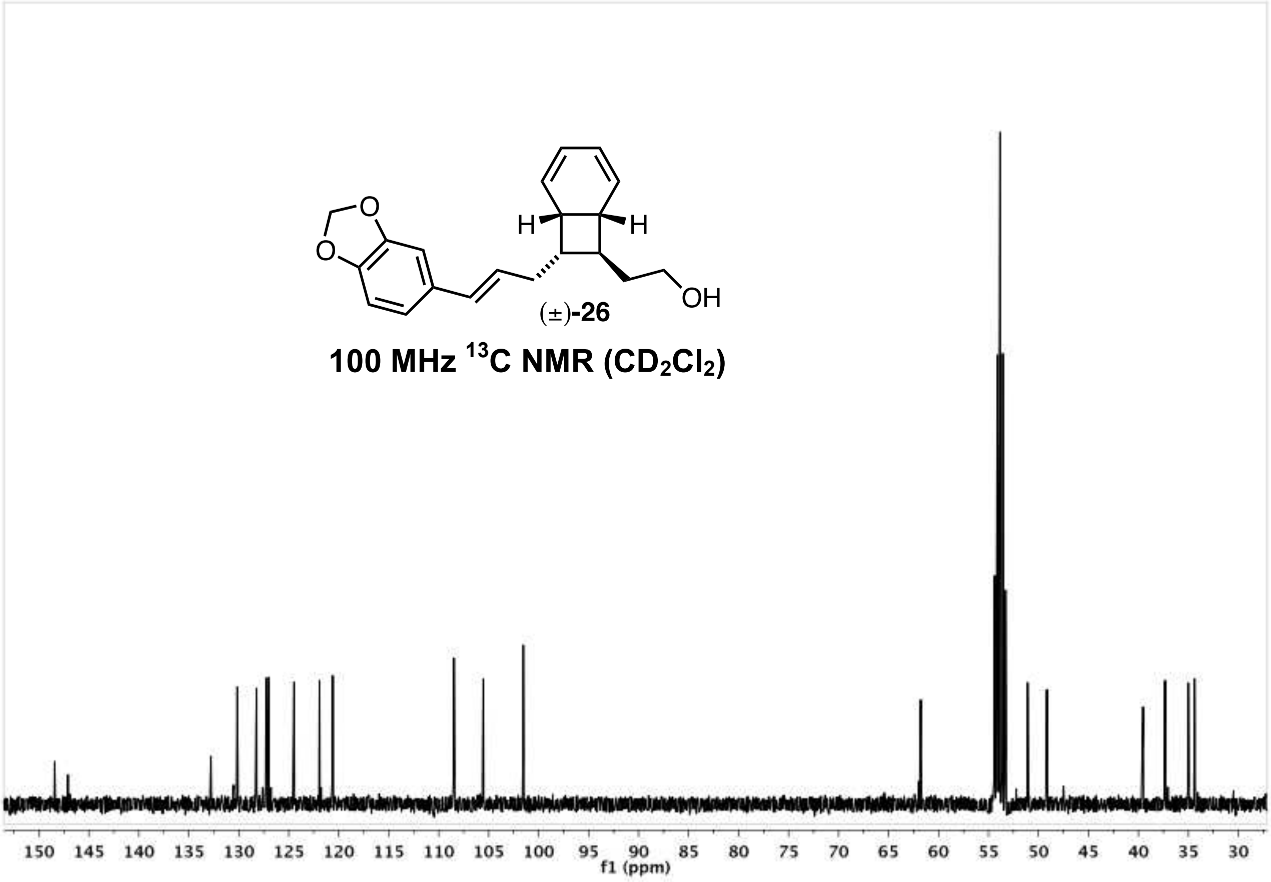
H
H
OH
O
O
(±)-26
100 MHz 13C NMR (CD2Cl2)
150 145 140 135 130 125 120 115 110 105 100 95 90 85 80 75 70 65 60 55 50 45 40 35 30
f1 (ppm)

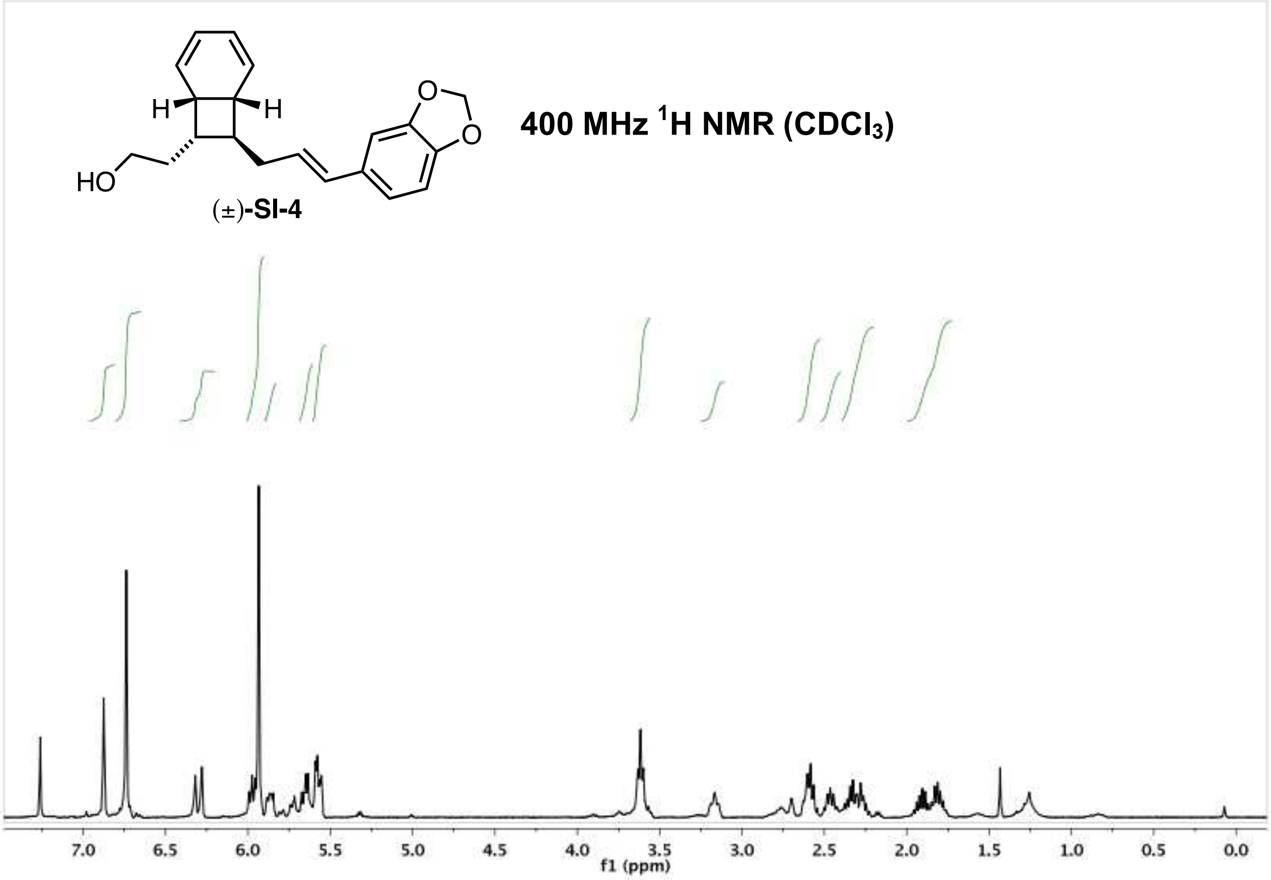

H
H
O
O
HO
(±)-SI-4
400 MHz ¹H NMR (CDCl₃)
f1 (ppm)

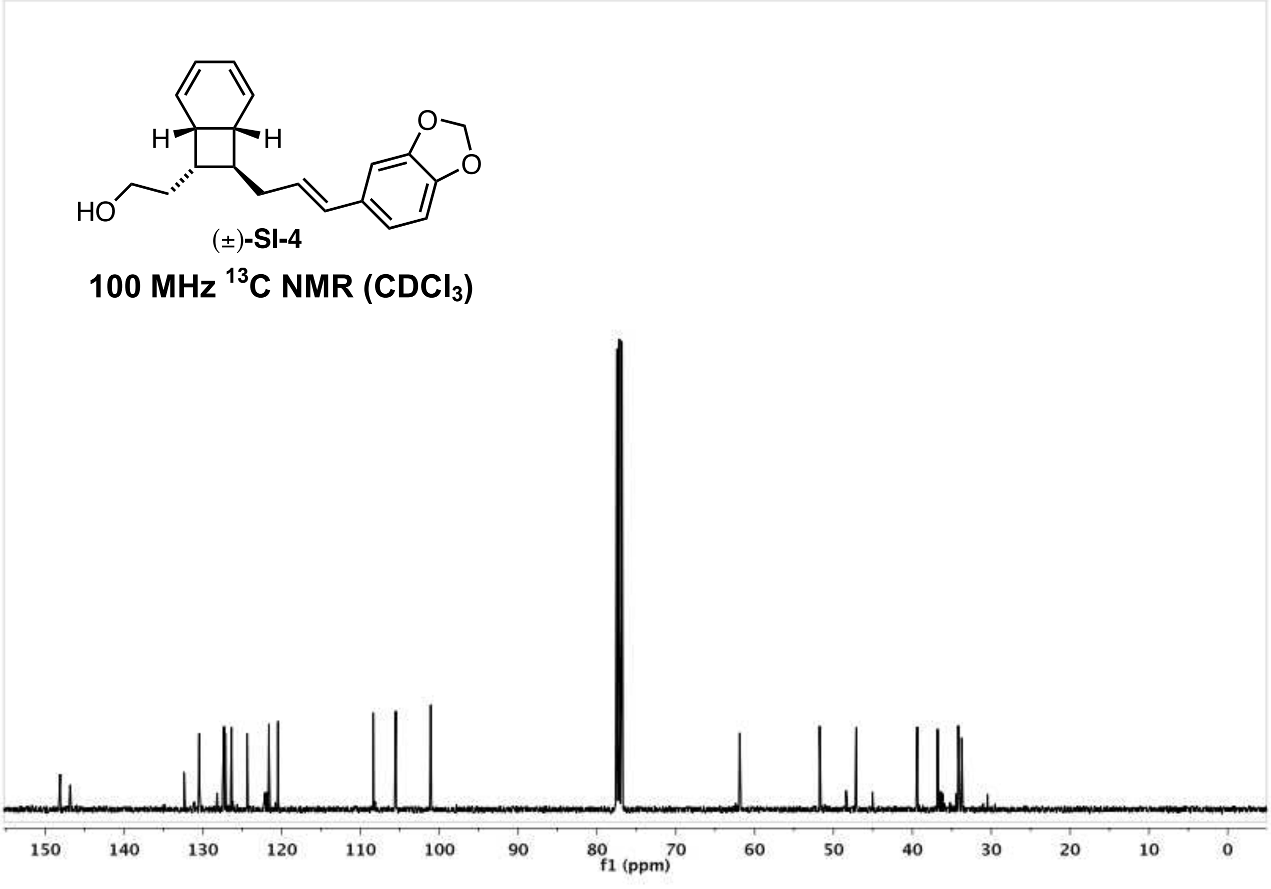

H
H
O
O
HO
(±)-SI-4
100 MHz ¹³C NMR (CDCl₃)
f1 (ppm)
